# The *Cyprinodon variegatus* genome reveals gene expression changes underlying differences in skull morphology among closely related species

**DOI:** 10.1186/s12864-017-3810-7

**Published:** 2017-05-30

**Authors:** Ezra S. Lencer, Wesley C. Warren, Richard Harrison, Amy R. McCune

**Affiliations:** 1 Department of Ecology and Evolutionary Biology, Cornell University, Ithaca, NY 14850 USA; 2 McDonnell Genome Institute, Washington University School of Medicine, St Louis, MO 63108 USA

**Keywords:** Craniofacial, Transcriptomics, Development, Genome, Skull, Pupfish, Evolution

## Abstract

**Background:**

Understanding the genetic and developmental origins of phenotypic novelty is central to the study of biological diversity. In this study we identify modifications to the expression of genes at four developmental stages that may underlie jaw morphological differences among three closely related species of pupfish (genus *Cyprinodon*) from San Salvador Island, Bahamas. Pupfishes on San Salvador Island are trophically differentiated and include two endemic species that have evolved jaw morphologies unlike that of any other species in the genus *Cyprinodon*.

**Results:**

We find that gene expression differs significantly across recently diverged species of pupfish. Genes such as *Bmp4* and calmodulin, previously implicated in jaw diversification in African cichlid fishes and Galapagos finches, were not found to be differentially expressed among species of pupfish. Instead we find multiple growth factors and cytokine/chemokine genes to be differentially expressed among these pupfish taxa. These include both genes and pathways known to affect craniofacial development, such as Wnt signaling, as well as novel genes and pathways not previously implicated in craniofacial development. These data highlight both shared and potentially unique sources of jaw diversity in pupfish and those identified in other evolutionary model systems such as Galapagos finches and African cichlids.

**Conclusions:**

We identify modifications to the expression of genes involved in Wnt signaling, Igf signaling, and the inflammation response as promising avenues for future research. Our project provides insight into the magnitude of gene expression changes contributing to the evolution of morphological novelties, such as jaw structure, in recently diverged pupfish species.

**Electronic supplementary material:**

The online version of this article (doi:10.1186/s12864-017-3810-7) contains supplementary material, which is available to authorized users.

## Background

A central goal of evolutionary biology is to understand the origins of phenotypic diversity. Critical to this task is elucidating how new phenotypic variation is produced during the early stages of species diversification. It is now widely appreciated that modified gene expression often underlies the origins of new variation at both deep and shallow phylogenetic scales [1–5]. Studies have largely focused on identifying how the expression of conserved genes are modified in different taxa. However, until recently, a challenge has been to identify additional sets of genes that may contribute to variation in phenotypes of interest. This is especially important for complex traits where phenotypic variation available to selection may be produced through interactions of multiple genes as well as environmental factors.

Skull morphology is an ecologically critical complex trait that varies widely across vertebrate taxa, and fishes offer a great diversity of skull and jaw morphology that are both functionally important and relatively accessible to study [6–10]. Model organisms such as zebrafish or medaka lack the phenotypic diversity of interest, but non-model organisms like cichlid fishes or pupfishes display this diversity and are also easy to rear in the lab [9, 11–13]. From a developmental perspective, specification and differentiation of skeletal head elements depends on complex interactions between the brain, facial epithelium, neural crest derived mesenchyme, and head endoderm during embryonic development [14–17]. Given the complexity of skull development, it is often thought that the enormous diversity of vertebrate skull forms could be produced through tweaking a conserved skull developmental program in different ways [10, 11, 18, 19]. Our understanding of how skull morphological diversity is produced in wild taxa is still largely limited to work on Galapagos finches and African cichlids. Amazingly, early work in both finches and cichlids showed that differences in jaw morphology is associated with altered expression of the same genes, *Bmp4* and calmodulin [11, 20–22]. However, sources of jaw phenotypic variation can be unique. Ongoing work in Caribbean bullfinches, close relatives of Galapagos finches, indicated that modification to the expression of different genes underlie jaw diversity among these taxa [19]. Other mechanisms, including roles for hedgehog signaling and Wnt signaling, have been proposed in different taxa [16, 23–29]. Thus a major next step is to both understand how and in what ways the genetic sources of phenotypic diversity in skull form vary across clades, as well as to identify additional genes and potential regulatory interactions that link gene expression to alterations in cell behavior that ultimately produce morphological variation.

**Fig. 1:**
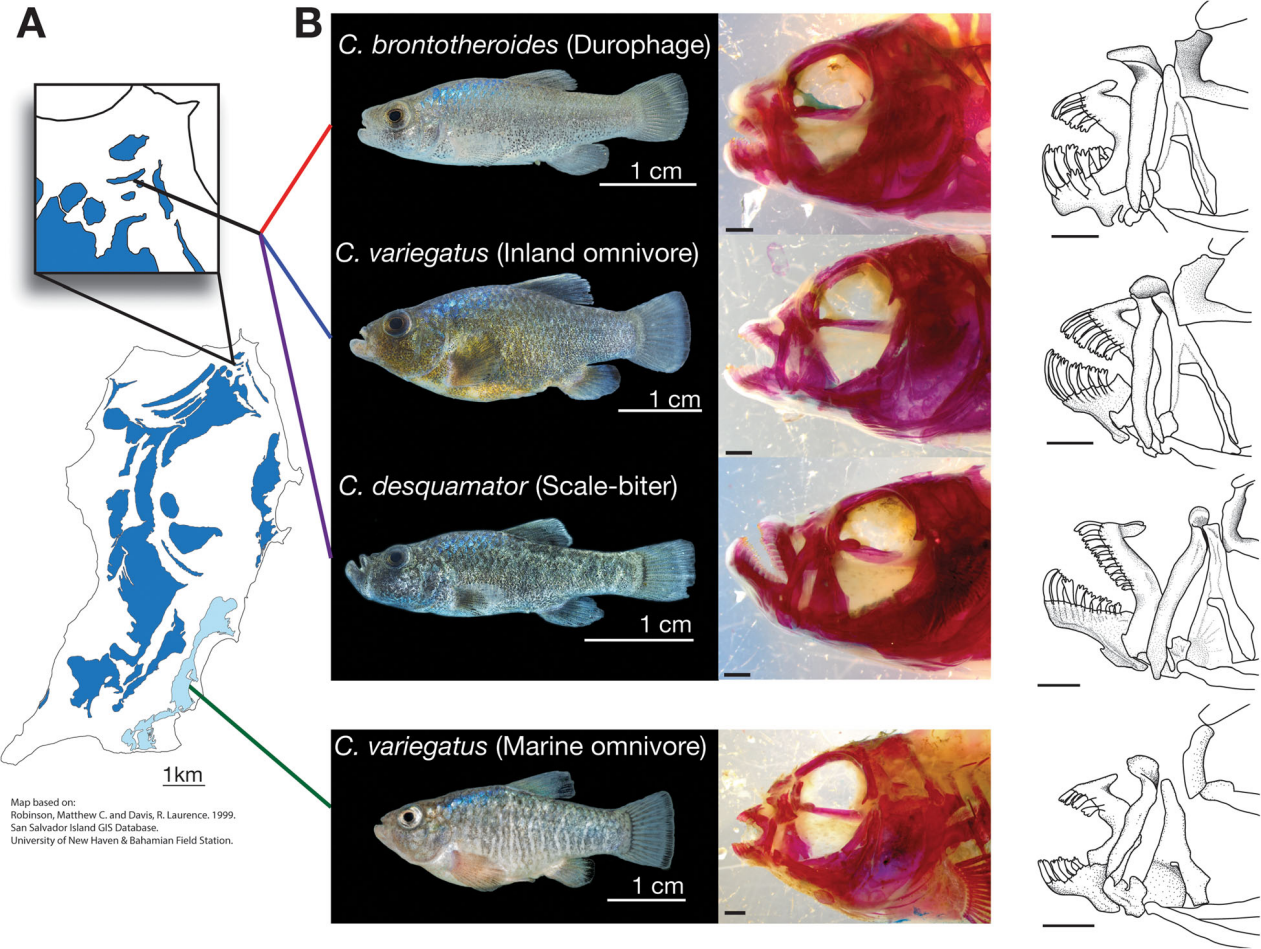
Morphology and sampling locations of San Salvador Island pupfishes. **a** Map of San Salvador Island indicating sites where wild parental fish used in the experiment were sourced. Wild fish of two endemic species, *C. brontotheroides* and *C. desquamator*, were collected from Crescent Pond at the north end of the island (inset). Two distinct populations of a third species, *C. variegatus*, were sourced from both Crescent Pond and a marine lagoon at the south end of the island. **b** Representative images illustrate the phenotypes of these 3 wild caught species. Shown are adult male fish in lateral view and the accompanying images of the skulls of these same fish stained with alizarin to visualize bone (*red*). Camera lucida drawings of cleared and stained fish highlight species differences in jaw morphology. Note the elongation of the jaws in the scale-biter, *C. desquamator*, the robust short jaws of the durophage, *C. brontotheroides*, and the similar jaw morphology of the inland and marine forms of the omnivore, *C. variegatus*. Scale bars = 1 mm unless noted. Map of San Salvador is based on [96]

Here, we use RNA-seq to identify a set of genes that may contribute to striking differences in jaw morphology among three ecologically differentiated pupfish species (genus *Cyprinodon*) from San Salvador Island Bahamas (Fig. 1). One of these island species is a population of the widespread *C. variegatus*, believed to have the pleisiomorphic jaw morphology for the group, while the other two species are endemic to San Salvador Island and exhibit unique jaw morphologies among the ~50 species of *Cyprinodon* [12, 13, 30, 31].

Our previous research indicates that species differ in the relative growth rates of individual bones of the skull during larval and juvenile growth [13]. Modifications to growth rates have long been proposed as a mechanism by which morphological diversity may be produced [32, 33], however, there is little known about how growth is modified at a molecular level, especially among wild taxa.

In pupfishes, altered growth of jaw bones might be due to altered gene expression during embryonic development as occurs in cichlids and finches, or it could be due largely to altered morphogenetic growth processes during post-hatching development. To investigate, we characterize gene expression among species at four developmental stages corresponding to major periods of skull differentiation and growth during both embryonic and post-hatching life stages. We sequenced and assembled the genome of the pupfish, *C. variegatus*, to serve as our reference in these studies. Our data identify a number of transcription factors, growth factors, and bone cell stimulatory molecules differentially expressed at embryonic and larval periods that may be related to differences in skull morphology among species of San Salvador pupfishes. Here we report on the *Cyprinodon* genome and the results from the RNA-seq study, revealing potential sources of skull morphological variation in pupfishes.

## Methods

### Study system

San Salvador pupfish species differ dramatically in cranial morphology and trophic ecology [12, 13]. One species, considered a population of the widespread *C. variegatus*, is an omnivore that exhibits the likely ancestral morphology [12, 34, 35]. The two other species on the island are endemic and exhibit unique jaw morphologies relative to all other *Cyprinodon* [13, 30, 36]. A scale-biter, *C. desquamator*, uses its enlarged upturned jaws to feed on the scales and body slime of other pupfishes (Fig. 1). The durophage, *C. brontotheroides,* feeds on hard-shelled prey such as snails, and has small robust jaws that are nested underneath maxilla and nasal bone extensions (Fig. 1). All three species occur in sympatry in a number of inland saltwater lakes on San Salvador Island. These inland lakes are not connected with the ocean. A population of *C. variegatus* also occurs in a mangrove estuary on the southern end of the island, and this hardy species is widespread on the North Atlantic coast from Massachusetts to Florida [37, 38]. Previous phylogenetic studies have suggested the mangrove estuarine population of *C. variegatus* and the inland lacustrine populations are genetically distinct despite their morphological similarity and taxonomic identity [31, 39]. For simplicity, in this paper, we will use informal names for these taxa descriptive by trophic ecology and/or habitat. We thus refer to *C. desquamator* as the scale-biter and *C. brontotheroides* as the durophage*.* Sympatric with these two species are the inland *C. variegatus* or the “inland omnivore.” The marine estuarine population of *C. variegatus* is simply the “marine omnivore.”

Morphological differences among species of pupfishes arise from differential growth rates of individual bones [13]. Oral jaw bones in the scale-biter grow at significantly faster rates during post-hatching growth than either of the other two species. In contrast, oral jaw bones of the durophage increase in size at significantly slower rates than either of the other two species during this same period. Changes to the relative growth rates of these individual jaw bones affect not only the adult size and shape of the bones, but also the overall skull shape, through the relative placement and interconnections of individual bones. For example the relatively small robust jaws of the durophage means that the jaws are positioned underneath the maxilla and nasal bones, giving the skull a very different appearance than in other species (Fig. 1). Measurable morphological differences emerge through juvenile growth, and by 17 days post fertilization (dpf), juvenile fish of each species have measurable differences in relative jaw size. However, these differences in growth rates during post-hatching stages could arise from either embryonic specification of jaw structures prior to hatching or from modifications solely to post-hatching growth.

### Genome sequencing and annotation

We sequenced and annotated the genome of *C. variegatus*. DNA used for sequencing the *C. variegatus* genome was obtained from a tissue sample of a single female identified as N-32, collected in 2010 at Navarre, FL, USA and provided to us by Diane Nacci at the EPA. Sequences of 100 bp length were generated on an Illumina HiSeq2500 instrument. Sequences were assembled according to default parameter recommendations provided in the AllPaths-LG assembler [40]. This model requires ~40× sequence coverage from each of overlapping (200 bp fragment size) and 3 kb Illumina paired-end (PE) reads and 10× of 8 kb PE Illumina reads. In the *C_variegatus-*1.0 assembly (NCBI) we removed contaminating contigs, trimmed vector sequence in the form of X’s, and ambiguous bases in the form of N’s in the sequence data. All scaffolds (singletons) and contigs within scaffolds that were 200 bp and less in length were removed from the assembly.

Gene annotation of the *C. variegatus* reference genome (C._variegatus-1.0) was completed according to previously established NCBI procedures (https://www.ncbi.nlm.nih.gov/genome/annotation_euk/process/).

### Animal husbandry and breeding for RNA-seq study

Wild caught *Cyprinodon* pupfishes from San Salvador Island, Bahamas, were maintained at Cornell University in 5 parts per thousand (ppt) brackish water at a constant temperature of 27 °C and a 14 h light/10 h dark schedule. Male-female pairs of wild caught fish were allowed to breed undisturbed for 1 h, after which clutches of eggs were collected and reared in petri-dishes with daily water changes. Larvae were transferred to 3 gal zebrafish rack tanks after hatching, which typically occurred between 7 to 8 days post fertilization (dpf). Hatched fish were fed daily live brine shrimp ad libitum beginning on 8 dpf, and water was changed every other day. All wild collections, animal husbandry, and procedures were approved by Cornell IACUC, protocol number 2011–0045 to ESL and ARM. Field research was conducted under Bahamas Environment, Science & Technology permit issued on November 10, 2012 and Export Permit 23/2013 issued on February 4, 2013.

### Sampling and dissections for RNA-seq

Full-sib clutches were sampled at 48 h post fertilization (hpf) corresponding to medaka embryonic stage 26–27 (*Cyprinodon* stage 26 from Lencer in prep.; head development similar to approx. Early Pharyngula Period of zebrafish) [41], 96 hpf corresponding to medaka embryonic stage 34–35 (*Cyprinodon* stage 30 from Lencer in prep.; head development similar to approx. Early Hatching Period of zebrafish) [41], 8 dpf (hatching larvae), and 15 dpf (juvenile fish). The stages sampled here cover a wide span of developmental time during which cell types and organs are differentiating. To account for this and to make samples as comparable across stages as possible we performed slightly different dissections at each stage. For example, we excluded eye and brain tissue from latter stages in order to remove these transcriptionally active organs. Dissections were performed similarly for all species, and we focus our comparisons among different taxa sampled at the same stage (see below). Fish were euthanized with an overdose of MS-222. For embryonic stages, head tissue was dissected away from the body and placed in RNAlater (Thermo Fisher Scientific) for long term storage. The 48 hpf samples were dissected by removing the body away from the yolk and heart using forceps, and then by removing the entire head just posterior to the developing otic capsule. For 96 hpf fish, the body was dissected from the yolk and heart and the eyes were removed using forceps. The entire head of 96 hpf fish was then removed from the body just anterior to the pectoral fin buds.

Larval and juvenile fish (8 dpf and 15 dpf) were stored in RNAlater immediately after euthanizing, and dissections were conducted in RNAlater at a subsequent date. The eyes and brain of larval fish were gently removed with forceps. The head was then removed from the body anterior to the pectoral girdle by gently pulling the heart posteriorly away from the head to separate the pharyngeal arches from the yolk, and by pulling gently along the pectoral girdle anterior edge to fully separate the head.

### RNA-seq library preparation

Four biological replicates were included for each species at each stage, where each biological replicate was produced from pools of the dissected heads of 10 full siblings sampled at the same stage. We used a different parental pair for each biological replicate of a given species at a given stage (total number of parental pairs across all stages are: durophage = 5, scale-biter = 6; inland omnivore = 4, marine omnivore = 5).

Total RNA was extracted using the TRIzol Plus RNA Purification Kit (Thermo Fisher Scientific) and checked for quality by running on an agarose gel. A subset of samples were quality checked for RNA integrity using an Agilent bioanalyzer.

Total RNA was treated with TURBO DNase (Thermo Fisher Scientific) followed by mRNA purification by processing through the NEBNext Poly(A) mRNA Magnetic Isolation Module twice (NEB). Isolated mRNA was used as library preparation material for Illumina sequencing using the NEBNext Ultra directional kit and NEBNext Multiplex Oligos for Illumina (NEB) as follows: fragmentation time was reduced to 4 min at 96 °C based on empirical results, final libraries were amplified using 15 PCR cycles, and final libraries post PCR were size selected using a two step AMPure bead isolation procedure (0.65×/0.15×, NEB manual). All libraries were quality checked by running on a 1.2% agarose gel. A subset of libraries were size checked on a Agilent bioanalyzer. All libraries showed a single peak of approximately 360–380 bp, indicating an insert size of around 240–260 bp.

Eight individually barcoded libraries (each lane had 2 libraries of a single species from each of the 4 taxa) were pooled and sequenced on a single Illumina lane (single end 150 bp sequencing Illumina HiSeq2500) for a total of 8 lanes and 64 samples (4 biological replicates for each of 4 taxa at each of 4 stages). A machine malfunction during the flow cell annealing step led to sequence quality dropping off after 60–100 bp in our final sequences. This machine was fixed and all libraries were re-sequenced (again single end 150 bp sequencing Illumina HiSeq2500). This second sequencing reaction produced 150 bp reads of high quality throughout the majority of reads. Running analyses separately on each sequencing run indicated that there were no major differences in results between the first and second sequencing runs, so reads from both sequencing runs were pooled and used for downstream analyses.

### Bioinformatic analysis of RNA-seq data

Reads were trimmed of adapter sequences and low quality regions using Trimmomatic [42], and all reads trimmed to a size shorter than 36 bp were discarded. Reads were aligned to the *Cyprinodon variegatus* genome (C. cyprinodon-1.0) using STAR aligner (version 2.5.1b), mapping only to annotated splicing junctions with a maximum mismatch of 3 bp per read [43]. Read counts per gene were generated using STAR quant mode, which is identical to the HTSeq union method of counting reads (personal observation; STAR manual). We observed no differences in mapping rates across taxa (Additional file 1: Table S1).

We built generalized linear models (GLM) in edgeR to analyze gene expression differences among pupfish taxa at each stage. Mitochondrial genes and genes annotated as pseudogenes were removed prior to analysis. We filtered low expressed genes to include only genes expressed at a level of at least 1 count per million (cpm; approx. 15–20 reads) in half of the samples of a given stage across all taxa, or at a level of 2 cpm in a quarter of the samples of a given stage across all taxa. With this filtering criteria, genes expressed in only a single taxon were maintained in our dataset. Changing the stringency of our filtering criteria has negligible consequences on our analyses and does not affect our major conclusions.

### Enrichment analyses

We identified 1-to-1 pupfish orthologs as the reciprocal best blast to zebrafish (NCBI, GRCz10). Gene names used in this paper correspond to ortholog assignments to zebrafish using this method. In cases where pupfish genes could not be assigned to a zebrafish ortholog we use the annotated pupfish gene identifier. Inspection of these genes indicated that many were paralogs of known genes. Teleosts, including pupfish and zebrafish, have undergone a whole genome duplication and we suspect that some paralogous gene copies could not be assigned as 1-to-1 orthologs using a reciprocal best blast approach. Orthology tables obtained from Zebrafish Model Organism (ZFIN) and Mouse Genome Informatics (MGI) databases were used to identify pupfish orthology relationships to mice and human genes.

We performed gene set enrichment analysis (GSEA, version 2–2.24) as implemented in the Broad Institute’s java command line program [44]. Genes were pre-ranked by either the log2-fold difference in expression values based on edgeR results, or in a separate series of analyses by each gene’s loadings on the first 3–4 principal component axes (see results). We tested for enrichment of gene sets in the GSEA hallmark sets v6 (50 gene sets) and the canonical pathways set v6 (1329 gene sets) using the classic scoring scheme and conducted 1000 permutations to determine significance. Genes without human identifiers were excluded from the analysis. Our results are similar when ranking genes by the mean log2 fold difference across all pairwise comparisons to a focal taxon, or by ranking genes based on significance.

For overrepresentation analysis, we used Blast2GO software to extract gene ontology (GO) annotations for genes based on BLAST similarity to UniProtKB/Swiss-Prot protein sequences. The package GOstats, as implemented in R, was used to perform enrichment analyses [45]. We additionally used DAVID and WebGestalt software to perform enrichment analyses using either the zebrafish reciprocal best blast gene ids (DAVID) or Human gene symbols (WebGestalt) as gene inputs [46, 47]. Results from these analyses were used heuristically to manually curate genes into functional categories [47, 48].

### Phylogenetic analysis

**Fig. 2:**
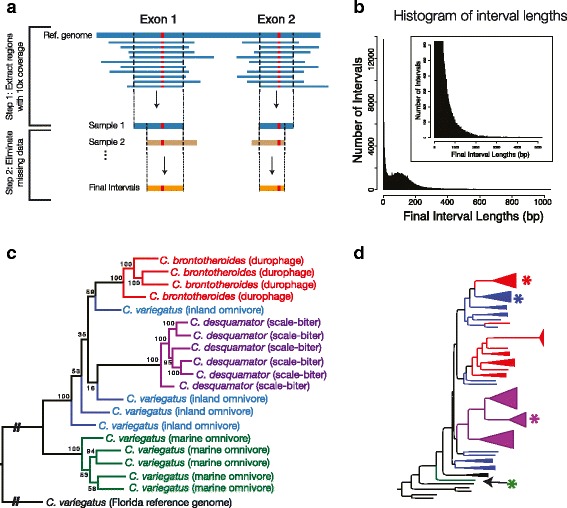
Phylogenetic relationships among species of pupfish from San Salvador Island. **a** Overview of bioinformatic methods to generate sequence data for phylogenetic analysis. Consensus genome sequences were generated for each parental pair by substituting SNP (*red bars*) allele assignments. Genomic intervals for use in phylogenetic analysis were selected based on contiguous regions that had a minimum coverage of 10 reads at every nucleotide position. We then further selected intervals across all parental pairs such that there was no missing data in our dataset. **b** The length distribution of genomic intervals used for phylogenetic reconstruction (from **a**). Inset shows intervals exceeding 1 kb in length. Many intervals are less than 10 bp long and some intervals are over 1 kb in length. We restricted our final interval set to regions that were longer than 50 bp and less than 1 kb. **c.** A maximum likelihood tree built from expressed exonic sequence data produced from the current study, and **d.** a maximum likelihood tree built from RAD-seq data, reproduced here from Martin and Feinstein [31, 50]. Note the overall congruence in the inferred phylogenetic relationships among taxa from both studies. In particular, both studies find a monophyletic San Salvador Island clade and that the marine omnivore population diverges prior to the speciation of endemic San Salvador taxa. Stars in **d.** indicate samples from the same populations as those of the current study

SNPs were called from RNA-seq reads using the GATK haplotype caller following the recommended “Best Practices” for RNA-seq data. Reads were mapped to the *C. variegatus* genome using STAR aligner in a two pass method that uses the predicted splicing junctions from the first pass as the annotated splicing junctions for the second mapping pass. Duplicate reads were marked using Picard Tools, spiced reads were split using the GATK splitNcigar tool, and SNPs were called using the GATK haplotype caller including all libraries and excluding soft clipped bases. SNPs were filtered using the GATK Variant Filtration Tool (Fischer Strand >30, > 3 SNPs per 35 bp window, Quality by depth < 2). We used vcftools to further filter all indels, SNPs with a depth less than 10, and any missing data. We used bcftools to extract the consensus sequence for each replicate, and used bedtools to extract only those regions that had a coverage of at least 10 reads at all bases and a contiguous length of between 50 bp to 1 kb. These regions were concatenated to produce ~36 Mb of both variant and invariant exonic sequence (Fig. 2a,b).

We used RAxML to infer phylogenetic relationships among species. Tips in the phylogeny refer to parental pairs used in our experiment. A single durophage pair was excluded because we only had data for one stage. Sequence alignments were divided into 14 partitions using the k means algorithm as implemented in PartitionFinder [49]. We applied a GTR+ gamma model, and conducted 500 bootstraps to estimate node support. We found similar tree topologies when running our data as a single partition and when applying a GTRCAT model in RAxML indicating that our phylogenetic estimation from our data is robust to different models (Additional file 2: Figure S1).

## Results

### Genome assembly and gene annotation

Total assembled sequence coverage of Illumina sequences for genome assembly were ~81× using a genome size estimate of 1.0 Gb. The C._variegatus-1.0 assembly (GCA_000732505.1 accession number) comprises a total of 9259 scaffolds with an N50 scaffold length over 835 Kb (N50 contig length was 20.8 Kb). The total assembled size is 899 Mb excluding gaps.

Gene annotation of the C._variegatus-1.0 reference utilizing the NCBI pipeline generated 23,373 protein-coding genes and 1010 non-coding genes. A full report of this predicted gene set is given at https://www.ncbi.nlm.nih.gov/genome/annotation_euk/Cyprinodon_variegatus/100/.

### Phylogenetic relationships among San Salvador pupfishes

Phylogenetic relationships among species of pupfishes were determined using SNPs from the RNA-seq dataset called via the GATK pipeline and alignment of reads to the C._variegatus-1.0 reference. Of 267,263 variable sites, 202,778 were variable among the Bahamian taxa. The other 64,485 sites were only variable between the San Salvador Island samples in our study and the reference genome sequence reflecting the outgroup status of the Florida population from which our reference is derived. From these data we concatenated ~36 Mb of both variant and invariant expressed exonic sequence data that was used for phylogenetic inference (Fig. 2a,b).

A maximum likelihood (ML) tree estimated from our concatenated dataset using RAxML placed the marine omnivore population as an outgroup to a monophyletic San Salvador clade (Fig. 2c). This agrees with previous studies based on anonymous genomic loci identified from a RAD-seq dataset (Fig. 2d) [31, 39, 50]. Thus, despite the marine omnivore and inland omnivore being morphologically similar and sharing taxonomic identity, mounting evidence indicates that the marine omnivore is an outgroup to an endemic San Salvador clade, which includes all three trophic forms.

Among the three inland trophic forms of pupfish (scale-biter, durophage, and inland omnivore), we resolved the durophage and scale-biter as monophyletic in concordance with previous studies [31, 39]. However the inland omnivore is paraphyletic in our ML tree, and bootstrap support for nodes resolving inland omnivore samples tend to be low. The durophage and scale biter comprise less than 10% of all fish in any given lake (personal observation) [12, 35], and demographic processes may partially account for why we resolve these taxa as monophyletic in our tree. Paraphyly of the inland omnivore may reflect both incomplete lineage sorting and ongoing introgression among inland taxa [31, 39], or simply that the durophage and scale biter were derived from different populations of the inland omnivore. Low bootstrap support at inland omnivore nodes may also suggest the presence of a hard polytomy at the root of the San Salvador Island clade.

### Gene expression divergence among Taxa

**Fig. 3:**
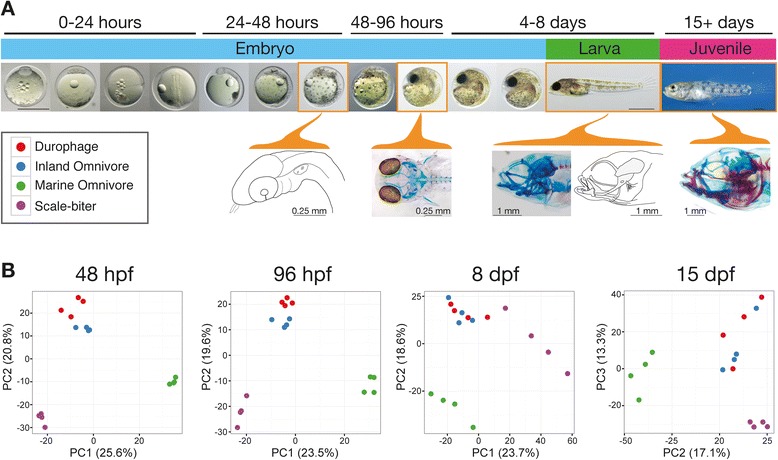
Gene expression patterns differ among species at all sampled stages. **a** Overview of pupfish development. The four developmental stages sampled in the current study are outlined with orange boxes. Camera lucida drawings and photos of fish stained for cartilage (*blue*) and bone (*red*) show head morphology at each of the sampled stages. At 48 hpf, fish resemble the pharyngula stage of zebrafish with migratory neural crest cells aggregated in the undifferentiated pharyngeal arches. By 96 hpf, the neurocranium and jaws first stain positive for cartilage (*blue*). Hatching 8 dpf larval fish have a mostly cartilaginous skull, but note the early ossification of dermal jaw bones that are highlighted in the camera lucida drawing. Morphological differences among pupfish jaws can be first measured in 15 dpf juvenile fish during a period of growth and increased bone deposition. **b** Species are separated by gene expression patterns along the first 2–3 principal component axes indicating a major effect of species in our dataset. A single PC axis typically separates the marine population from all three San Salvador taxa mirroring phylogenetic relationships from Fig. 2, while the inland omnivore samples tend to be more similar to the durophage samples than to samples from the morphologically similar marine omnivore population

Using RNA-seq, we measured gene expression in the heads of each of the four taxa at the following four developmental stages: (1) 48 h post fertilization (hpf) analogous to the zebrafish pharyngula period, (2) 96 hpf corresponding to an embryonic period when jaw and neurocranial cartilages are forming, (3) 8 days post fertilization (dpf) corresponding to larval hatching when jaw elements are similar between species, and (4) 15 dpf during a period of juvenile growth when measurable differences in jaw size among species emerge as a consequence of differential growth of individual bony elements (Fig. 3a) [13].

Sequencing produced 2.7 billion reads total after filtering (24.3–54.4 million reads per sample). Mapping rates to the *C. variegatus* genome using STAR aligner exceeded 90% of reads being uniquely mapped for all samples, and we observed no differences in mapping rates among taxa (Additional file 1: Table S1).

Gene expression varied dramatically across developmental stage owing to true differences in tissue development and our slightly different method of dissections at different stages in an attempt to make the tissue samples as comparable as possible (Additional file 3: Figure S2). Within a stage, as expected, expression levels among libraries derived from all four taxa were highly correlated (Pearson’s *r* > 0.9). Because our primary interest is to understand differences among taxa, we restrict our subsequent analyses of differential expression to comparisons among taxa at the same developmental stage.

Gene expression patterns clearly show separation by taxon along the first 3 principal component axes (PC) for all stages (Fig. 3b; Additional file 4: Figure S3). Nearly half of the total variance in gene expression levels among samples at each stage is attributable to differences among taxa. Inland omnivore samples grouped at all four stages with the durophage samples rather than with the taxonomically and morphologically similar marine omnivore samples. At each stage, a single PC axis separates the marine omnivore population from all three inland San Salvador taxa mirroring the inferred phylogenetic relationships from our ML tree. Thus morphological similarity and gene expression divergence at a transcriptomic scale appear to be decoupled. This pattern would fit a model where either only slight modifications and/or modifications to the expression of only a small proportion of genes contribute to morphological differentiation. This pattern may also reflect ongoing introgression among the inland omnivore and the durophage taxa [31, 39], as well as the accumulation of gene expression differences with time since common ancestry.

### Gene set enrichment suggests modifications to conserved cellular processes

We used GSEA to test for enrichment of conserved cellular processes among the genes differentially expressed among taxa. Our conclusions are largely congruent across analyses conducted on the hallmark collection and canonical pathways collection, and so we confine our discussion to results from enrichment of hallmark gene sets and direct interested readers to supplementary tables for more detailed results of canonical pathways (Additional file 5: Table S2, Additional file 6: Table S3, Additional file 7: Table S4, Additional file 8: Table S5, Additional file 9: Table S6 and Additional file 10: Table S7).

We first tested for enrichment of conserved cellular processes along each of the PC axes at each stage by ranking genes based on each gene’s loadings on a PC axis (Table 1; Additional file 5: Table S2 and Additional file 6: Table S3). As an example, PC1 at 48 hpf largely distinguishes the marine omnivore samples from all three of the inland taxa (Fig. 3b). Genes with positive loadings on this axis were enriched for cell cycle related processes such as E2F Targets and Myc Targets, while genes with negative loadings on this axis were enriched for genes involved in the epithelial to mesenchymal transition and KRAS signaling (Table 1; Additional file 5: Table S2). The first several PC axes that distinguish taxa at each stage (see Fig. 3b; Additional file 4: Figure S3) show repeated evidence for enrichment of genes related to cell cycle control, myogenesis, protein secretion, metabolism, the estrogen response, the inflammation response, and genes involved in the epithelial to mesenchymal transition. We observed enrichment for a number of signaling processes including TNF alpha/NF-kB, Interferon alpha and gamma responses, IL-6/Jak/Stat signaling, KRAS signaling, Myc signaling, and to a lesser extent Wnt, Tgf-B, and hedgehog signaling. Results from GSEA enrichment analysis of canonical pathways were congruent with results based on the hallmark gene sets (Additional file 6: Table S3).

**Table 1:** Enrichment (GSEA) of top Hallmark Gene Sets along principal component axes for each stage

48 Hours Post Fertilization		96 Hours Post Fertilization		8 Days Post Fertilization		15 Days Post Fertilization	
**PC1** **(Separates Marine From Inland Taxa)**		**PC1** **(Separates Marine From Inland Taxa)**		**PC1** **(Separates Scale-Biter From Taxa)**		**PC2** **(Separates Marine From Inland Taxa)**	
Name	NES	Name	NES	Name	NES	Name	NES
*Positive Loadings:*		*Positive Loadings:*		*Positive Loadings:*		*Positive Loadings:*	
**E2F Targets**	**2.84**	**Interferon Gamma Resp.**	**2.81**	**Uv Response Dn**	**2.53**	**Myogenesis**	**2.99**
**G2M Checkpoint**	**2.73**	**Interferon Alpha Response**	**2.14**	**Il2 Stat5 Signaling**	**2.36**	**Il2 Stat5 Signaling**	**2.88**
**Mitotic Spindle**	**2.17**	**Xenobiotic Metabolism**	**2.14**	**Tnfa Signaling Via Nfkb**	**2.35**	**Uv Response Dn**	**2.86**
**Protein Secretion**	**2.14**	**Bile Acid Metabolism**	**1.98**	**Kras Signaling Dn**	**2.29**	**Inflammatory Response**	**2.64**
**Myc Targets V1**	**1.86**	**Protein Secretion**	**1.82**	**Allograft Rejection**	**2.18**	**Allograft Rejection**	**2.57**
**Dna Repair**	**1.74**	**Oxidative Phosphorylation**	**1.80**	**P53 Pathway**	**2.10**	**Kras Signaling Dn**	**2.54**
**Xenobiotic Metabolism**	**1.54**	**E2F Targets**	**1.73**	**Inflammatory Response**	**1.99**	**Hedgehog Signaling**	**2.05**
**Interferon Alpha Response**	**1.51**	**Glycolysis**	**1.66**	**Apical Junction**	**1.94**	**Apical Junction**	**2.04**
**Estrogen Response Late**	**1.50**	**Il6 Jak Stat3 Signaling**	**1.66**	**Kras Signaling Up**	**1.83**	**Coagulation**	**2.01**
*Negative Loadings:*		*Negative Loadings:*		*Negative Loadings:*		*Negative Loadings:*	
**Epith. Mesench. Trans.**	**−3.07**	Tnfa Signaling Via Nfkb	−1.81	**E2F Targets**	**−8.13**	**Myc Targets V1**	**−5.97**
**Coagulation**	**−2.12**	**Myc Targets V1**	**−1.74**	**G2M Checkpoint**	**−6.50**	**E2F Targets**	**−5.78**
**Kras Signaling Dn**	**−1.80**	**Epith. Mesench. Trans.**	**−1.70**	**Myc Targets V1**	**−5.56**	**G2M Checkpoint**	**−4.91**
**Oxidative Phosphorylation**	**−1.63**	**Apical Junction**	**−1.63**	**mTORc1 Signaling**	**−4.51**	**Oxidative Phosph.**	**−4.38**
**Peroxisome**	**−1.61**	**Kras Signaling Up**	**−1.62**	**Oxidative Phosph.**	**−3.06**	**Myc Targets V2**	**−4.03**
Inflammatory Response	−1.53	Androgen Response	−1.48	**Dna Repair**	**−2.87**	**mTORc1 Signaling**	**−3.59**
Il2 Stat5 Signaling	−1.29	Kras Signaling Dn	−1.29	**Myc Targets V2**	**−2.84**	**Dna Repair**	**−3.55**
Myc Targets V2	−1.25	Uv Response Dn	−1.23	**Unfolded Protein Resp.**	**−2.54**	**Fatty Acid Metabolism**	**−2.91**
Pancreas Beta Cells	−1.25	Notch Signaling	−1.19	**Mitotic Spindle**	**−2.50**	**Unfolded Protein Resp.**	**-2.44**
**PC2** **(Separates Taxa By Morphology)**		**PC2** **(Separates Taxa By Morphology)**		**PC2** **(Separates Marine From Inland Taxa)**		**PC3** **(Separates Scale-Biter From Taxa)**	
Name	NES	Name	NES	Name	NES	Name	NES
*Positive Loadings:*		*Positive Loadings:*		*Positive Loadings:*		*Positive Loadings:*	
**Myc Targets V1**	**3.98**	**Myogenesis**	**2.92**	**E2F Targets**	**6.93**	**Kras Signaling Dn**	**2.86**
**Oxidative Phosph.**	**3.10**	**Kras Signaling Up**	**2.27**	**Myc Targets V1**	**5.41**	**Uv Response Dn**	**2.64**
**Dna Repair**	**2.83**	**Interferon Gamma Resp.**	**2.21**	**G2M Checkpoint**	**4.95**	**Myogenesis**	**2.60**
**E2F Targets**	**2.50**	**Coagulation**	**1.90**	**Myc Targets V2**	**3.80**	**Epith. Mesench. Trans.**	**2.55**
**Myc Targets V2**	**2.48**	**Protein Secretion**	**1.83**	**Dna Repair**	**3.07**	**Tgf Beta Signaling**	**2.11**
**G2M Checkpoint**	**2.03**	**Apical Junction**	**1.82**	**mTORc1 Signaling**	**3.04**	**Hedgehog Signaling**	**2.06**
**Mitotic Spindle**	**1.87**	**Uv Response Dn**	**1.79**	**Unfolded Protein Response**	**2.86**	**Apical Junction**	**2.00**
**Bile Acid Metabolism**	**1.66**	**Epith. Mesench. Trans.**	**1.79**	**Oxidative Phosphorylation**	**1.96**	**Bile Acid Metabolism**	**1.96**
mTORc1 Signaling	1.27	**Kras Signaling Dn**	**1.74**	**Pancreas Beta Cells**	**1.74**	**Wnt B-Catenin Signaling**	**1.91**
*Negative Loadings:*		*Negative Loadings:*		*Negative Loadings:*		*Negative Loadings:*	
**Hypoxia**	**−2.76**	**G2M Checkpoint**	**−3.33**	**Myogenesis**	**−3.90**	**E2F Targets**	**−5.20**
**Epith. Mesench. Trans.**	**−2.69**	**Myc Targets V1**	**−2.96**	**Tnfa Signaling Via Nfkb**	**−3.54**	**mTORc1 Signaling**	**−5.05**
**Estrogen Response Early**	**−2.28**	**E2F Targets**	**−2.75**	**Hypoxia**	**−3.07**	**Myc Targets V1**	**−4.85**
**Pi3K/Akt/mTOR Sig.**	**−1.87**	**Oxidative Phosphorylation**	**−1.99**	**Interferon Gamma Resp.**	**−2.86**	**G2M Checkpoint**	**−4.03**
**Protein Secretion**	**−1.76**	**Myc Targets V2**	**−1.95**	**Il6 Jak Stat3 Signaling**	**−2.82**	**Myc Targets V2**	**−3.76**
**Tnfa Signaling Via Nfkb**	**−1.73**	**Dna Repair**	**−1.85**	**Epith. Mesench. Trans.**	**−2.74**	**Dna Repair**	**-3.50**
**P53 Pathway**	**−1.63**	**Wnt B-Catenin Signaling**	**−1.72**	**Uv Response Dn**	**−2.66**	**Unfolded Protein Resp.**	**−2.77**
**Kras Signaling Up**	**−1.60**	**Unfolded Protein Response**	**−1.68**	**Kras Signaling Up**	**−2.62**	**Oxidative Phosph.**	**−2.29**
**Myogenesis**	**−1.57**	**mTORc1 Signaling**	**−1.52**	**P53 Pathway**	**−2.57**	**Cholesterol Homeostasis**	**−2.19**

Shown are the top 9 hallmark pathways for each PC axis at each stage. Gene sets in bold are significant at FDR ≤ 0.25. *NES* normalized enrichment

We next used GSEA to test for enrichment of gene sets in the genes over- or underexpressed in either the durophage or the scale-biter at each developmental stage relative to all other taxa by ranking genes based on the estimated log2 fold difference. Perhaps not surprisingly we found many of the same gene sets enriched when ranking genes by over- or underexpression as we found enriched when ranking genes by loadings onto the PC axes that distinguish samples by taxa (Tables 2 and 3; Additional file 7: Table S4, Additional file 8: Table S5, Additional file 9: Table S6 and Additional file 10: Table S7). We observed enrichment at every stage for gene sets suggesting modification to cell cycle regulation. In particular, genes underexpressed in the scale-biter at 48 hpf and 8 dpf are greatly enriched for functions related to cell cycle regulation and progression as further evidenced by enrichment of canonical pathways (Additional file 9: Table S6). We found enriched categories related to Myc signaling, KRAS signaling, fatty acid metabolism, adipogenesis, myogenesis, the epithelial to mesenchymal transition. Genes related to the extracellular matrix (e.g. matrisome) were significantly enriched in multiple comparisons (Additional file 9: Table S6 and Additional file 10: Table S7).

**Table 2:** Enrichment (GSEA) of top Hallmark Gene Sets for genes over- or underexpressed in the Scale-biter relative to all other taxa. Genes were pre-ranked by log2 fold change prior to analysis

**48 Hours Post Fertilization**							
*Overexpressed in Scale-biter:*				*Underexpressed in Scale-biter:*			
Name	NES	Pvalue	FDR	Name	NES	Pvalue	FDR
**Epith. Mesench. Trans.**	**2.80**	**0.00**	**0.00**	**E2F Targets**	**−3.60**	**0.00**	**0.00**
**Hypoxia**	**2.27**	**2.0E-03**	**0.01**	**Myc Targets V1**	**−3.23**	**0.00**	**0.00**
**Kras Signaling Dn**	**2.08**	**2.1E-03**	**0.02**	**Dna Repair**	**−3.06**	**0.00**	**0.00**
**Estrogen Resp. Early**	**1.87**	**0.01**	**0.07**	**G2M Checkpoint**	**−2.94**	**0.00**	**2.4E-04**
**Coagulation**	**1.86**	**0.01**	**0.06**	**Oxidative Phosph.**	**−2.60**	**0.00**	**8.8E-04**
**Tnfa Sig. via Nfkb**	**1.81**	**0.02**	**0.07**	**Myc Targets V2**	**−2.53**	**0.00**	**1.1E-03**
**Angiogenesis**	**1.64**	**0.04**	**0.14**	**Mitotic Spindle**	**-2.29**	**1.9E-03**	**3.6E-03**
**Myogenesis**	**1.64**	**0.03**	**0.12**	**Interferon Alpha Resp.**	**−1.83**	**0.01**	**0.04**
**P53 Pathway**	**1.58**	**0.06**	**0.14**	**Mtorc1 Signaling**	**−1.70**	**0.03**	**0.07**
**Kras Signaling Up**	**1.54**	**0.05**	**0.15**	**Fatty Acid Metabolism**	**−1.65**	**0.03**	**0.08**
**96 Hours Post Fertilization**							
*Overexpressed in Scale-biter:*				*Underexpressed in Scale-biter:*			
Name	NES	Pvalue	FDR	Name	NES	Pvalue	FDR
**Myc Targets V1**	**2.69**	**0.00**	**1.2E-03**	**Bile Acid Metabolism**	**−2.59**	**0.00**	**2.0E-03**
**G2M Checkpoint**	**1.99**	**2.0E-03**	**0.07**	**Myogenesis**	**−2.44**	**0.00**	**3.8E-03**
**Androgen Response**	**1.69**	**0.03**	**0.21**	**Interferon Gamma Resp.**	**−2.01**	**0.01**	**0.05**
**E2F Targets**	**1.69**	**0.03**	**0.16**	**Interferon Alpha Resp.**	**−1.75**	**0.02**	**0.15**
**Myc Targets V2**	**1.68**	**0.03**	**0.13**	**Inflammatory Response**	**−1.68**	**0.03**	**0.18**
Unfolded Protein Resp.	1.47	0.07	0.29	**Adipogenesis**	**−1.66**	**0.03**	**0.16**
Notch Signaling	1.44	0.10	0.28	**Protein Secretion**	**−1.54**	**0.06**	**0.24**
Heme Metabolism	1.40	0.11	0.29	**Xenobiotic Metabolism**	**−1.51**	**0.07**	**0.24**
Mtorc1 Signaling	1.25	0.18	0.47	Oxidative Phosphorylation	−1.43	0.10	0.30
Tnfa Signaling Via Nfkb	1.11	0.32	0.69	Kras Signaling Up	−1.41	0.10	0.28
**8 Days Post Fertilization**							
*Overexpressed in Scale-biter:*				*Underexpressed in Scale-biter:*			
Name	NES	Pvalue	FDR	Name	Nes	Pvalue	FDR
**Uv Response Dn**	**2.25**	**0.00**	**0.02**	**E2F Targets**	**−5.90**	**0.00**	**0.00**
**Il2 Stat5 Signaling**	**2.14**	**0.01**	**0.02**	**G2M Checkpoint**	**−4.88**	**0.00**	**0.00**
**Kras Signaling Dn**	**2.12**	**0.00**	**0.01**	**Myc Targets V1**	**−4.18**	**0.00**	**0.00**
**Tnfa Signaling Via Nfkb**	**1.80**	**0.02**	**0.07**	**Oxidative Phosph.**	**−3.54**	**0.00**	**0.00**
**Angiogenesis**	**1.59**	**0.05**	**0.17**	**Mtorc1 Signaling**	**−3.42**	**0.00**	**0.00**
**Estrogen Response Early**	**1.58**	**0.04**	**0.15**	**Mitotic Spindle**	**−2.59**	**0.00**	**1.7E-04**
**Notch Signaling**	**1.54**	**0.06**	**0.16**	**Fatty Acid Metabolism**	**−2.37**	**0.00**	**2.3E-03**
**P53 Pathway**	**1.45**	**0.09**	**0.21**	**Dna Repair**	**−2.22**	**0.00**	**0.01**
**Apical Junction**	**1.45**	**0.09**	**0.18**	**Interferon Alpha Resp.**	**−1.82**	**0.02**	**0.05**
**Inflammatory Response**	**1.42**	**0.10**	**0.19**	**Glycolysis**	**−1.79**	**0.02**	**0.06**
**15 Days Post Fertilization**							
*Overexpressed in Scale-biter:*				*Underexpressed in Scale-biter:*			
Name	NES	Pvalue	FDR	Name	NES	Pvalue	FDR
**Inflammatory Response**	**2.25**	**2.0E-03**	**0.02**	**Oxidative Phosph.**	**−5.63**	**0.00**	**0.00**
**Cholesterol Homeostasis**	**2.13**	**2.1E-03**	**0.02**	**Myc Targets V1**	**−3.70**	**0.00**	**0.00**
**Wnt B-Catenin Signaling**	**2.02**	**3.9E-03**	**0.03**	**Fatty Acid Metabolism**	**−2.52**	**0.00**	**0.00**
**Hedgehog Signaling**	**1.94**	**0.01**	**0.04**	**Adipogenesis**	**−2.50**	**0.00**	**2.3E-04**
**Pi3K Akt Mtor Signaling**	**1.94**	**0.01**	**0.03**	**Dna Repair**	**−2.46**	**0.00**	**1.9E-04**
**Allograft Rejection**	**1.83**	**0.02**	**0.05**	**E2F Targets**	**−2.32**	**0.00**	**2.6E-03**
**Uv Response Dn**	**1.72**	**0.02**	**0.08**	**Bile Acid Metabolism**	**-2.19**	**1.9E-03**	**0.01**
**Complement**	**1.70**	**0.03**	**0.07**	**Tnfa Signaling Via Nfkb**	**−1.71**	**0.02**	**0.09**
**Estrogen Response Early**	**1.50**	**0.08**	**0.16**	**Xenobiotic Metabolism**	**−1.55**	**0.06**	**0.17**
**Apical Junction**	**1.41**	**0.10**	**0.23**	**Spermatogenesis**	**−1.51**	**0.07**	**0.19**

Shown are the top 10 genesets shown for each analysis. Gene sets significant at FDR ≤ 0.25 shown in bold

*NES* Normalized Enrichment, *Pvalue* Nominal *P* value, *FDR* False Discover Rate (Q value)

**Table 3:** Enrichment (GSEA) of top Hallmark Gene Sets for genes over- or underexpressed in the Durophage relative to all other taxa. Genes were pre-ranked by log2 fold change prior to analysis

**48 Hours Post Fertilization**							
*Overexpressed in Durophage:*				*Underexpressed in Durophage:*			
Name	NES	Pvalue	FDR	Name	NES	Pvalue	FDR
Inflammatory Response	1.74	0.02	0.39	**E2F Targets**	**−2.77**	**0.00**	**0**
Pancreas Beta Cells	1.65	0.04	0.31	**Myc Targets V1**	**−2.42**	**0.00**	**2.74E-03**
**Uv Response Dn**	**1.62**	**0.04**	**0.23**	**G2M Checkpoint**	**-2.35**	**2.0E-03**	**0.01**
Mitotic Spindle	1.53	0.05	0.27	**Oxidative Phosphory.**	**−2.30**	**0**	**4.76E-03**
Epith.Mesench.Trans.	1.43	0.10	0.34	**Hypoxia**	**-2.15**	**2.0E-03**	**0.01**
Myogenesis	1.43	0.08	0.29	**Adipogenesis**	**−1.85**	**0.01**	**0.06**
Hedgehog Signaling	1.31	0.16	0.38	**Protein Secretion**	**−1.73**	**0.02**	**0.11**
Kras Signaling Up	1.17	0.26	0.57	**Pi3K/Akt/mTOR Sig.**	**−1.72**	**0.02**	**0.10**
Il2 Stat5 Signaling	1.12	0.30	0.59	**Fatty Acid Metabolism**	**−1.67**	**0.04**	**0.11**
Apical Junction	0.94	0.53	0.93	**Myc Targets V2**	**−1.64**	**0.04**	**0.12**
**96 Hours Post Fertilization**							
*Overexpressed in Durophage:*				*Underexpressed in Durophage:*			
Name	NES	Pvalue	FDR	Name	NES	Pvalue	FDR
**E2F Targets**	**2.01**	**0.01**	**0.12**	**Hypoxia**	**−2.42**	**0.00**	**0.01**
**Mitotic Spindle**	**1.95**	**0.01**	**0.08**	**Tnfa Signaling Via Nfkb**	**−1.94**	**0.01**	**0.09**
**Myogenesis**	**1.83**	**0.01**	**0.11**	**Protein Secretion**	**−1.77**	**0.01**	**0.15**
**Bile Acid Metabolism**	**1.73**	**0.03**	**0.15**	Oxidative Phosphorylation	−1.55	0.06	0.31
**Inflammatory Response**	**1.71**	**0.02**	**0.13**	Epith. Mesench.Trans.	−1.45	0.09	0.40
**Interferon Alpha Resp.**	**1.70**	**0.02**	**0.12**	Cholesterol Homeostasis	−1.34	0.14	0.53
**Myc Targets V1**	**1.54**	**0.06**	**0.22**	Il6 Jak Stat3 Signaling	−1.33	0.13	0.46
**Dna Repair**	**1.53**	**0.06**	**0.20**	Estrogen Response Late	−1.32	0.15	0.42
**Uv Response Dn**	**1.51**	**0.06**	**0.20**	Kras Signaling Up	−1.31	0.14	0.39
Pi3K/Akt/mTOR Sig.	1.39	0.12	0.30	Estrogen Early Resp.	−1.30	0.16	0.36
**8 Days Post Fertilization**							
*Overexpressed in Durophage:*				*Underexpressed in Durophage:*			
Name	NES	Pvalue	FDR	Name	Nes	Pvalue	FDR
**Myc Targets V1**	**3.23**	**0.00**	**0.00**	**Protein Secretion**	**-2.34**	**2.1E-03**	**0.01**
**E2F Targets**	**3.16**	**0.00**	**0.00**	**Coagulation**	**-2.16**	**1.9E-03**	**0.02**
**Allograft Rejection**	**2.89**	**0.00**	**0.00**	**Uv Response Dn**	**−1.83**	**0.02**	**0.11**
**Oxidative Phosph.**	**2.85**	**0.00**	**0.00**	**Estrogen Response Early**	**−1.80**	**0.03**	**0.10**
**Dna Repair**	**2.31**	**0.00**	**4.3E-03**	**Estrogen Response Late**	**−1.78**	**0.02**	**0.09**
**Myc Targets V2**	**1.90**	**4.1E-03**	**0.04**	**Hypoxia**	**−1.65**	**0.04**	**0.15**
**G2M Checkpoint**	**1.82**	**0.02**	**0.05**	**Kras Signaling Dn**	**−1.58**	**0.05**	**0.17**
**Mitotic Spindle**	**1.67**	**0.04**	**0.10**	**Notch Signaling**	**−1.57**	**0.05**	**0.16**
**ROS Pathway**	**1.61**	**0.04**	**0.12**	Cholesterol Homeostasis	−1.40	0.09	0.30
Interferon Gamma Resp.	1.40	0.11	0.26	Hedgehog Signaling	−1.38	0.11	0.28
**15 Days Post Fertilization**							
*Overexpressed in Durophage:*				*Underexpressed in Durophage:*			
Name	NES	Pvalue	FDR	Name	NES	Pvalue	FDR
**Myogenesis**	**3.59**	**0.00**	**0.00**	**G2M Checkpoint**	**−4.25**	**0.00**	**0.00**
**Allograft Rejection**	**3.54**	**0.00**	**0.00**	**E2F Targets**	**−4.05**	**0.00**	**0.00**
**Interferon Gamma Resp.**	**2.31**	**4.0E-03**	**0.01**	**Mtorc1 Signaling**	**−2.97**	**0.00**	**0.00**
**Inflammatory Response**	**2.16**	**0**	**0.01**	**Cholesterol Homeostasis**	**−2.69**	**0.00**	**2.2E-04**
**Bile Acid Metabolism**	**2.06**	**2.0E-03**	**0.01**	**Myc Targets V1**	**−2.31**	**0.00**	**3.5E-03**
**Il2 Stat5 Signaling**	**1.94**	**0.01**	**0.03**	**Oxidative Phosphory.**	**−2.31**	**0.00**	**2.9E-03**
**Interferon Alpha Resp.**	**1.85**	**0.01**	**0.04**	**Mitotic Spindle**	**-2.17**	**1.9E-03**	**0.01**
**P53 Pathway**	**1.82**	**0.01**	**0.04**	**Estrogen Response Late**	**−1.65**	**0.04**	**0.11**
**Uv Response Dn**	**1.74**	**0.02**	**0.06**	**Adipogenesis**	**−1.51**	**0.06**	**0.18**
**Kras Signaling Up**	**1.73**	**0.02**	**0.06**	**Fatty Acid Metabolism**	**−1.50**	**0.06**	**0.17**

Shown are the top 10 gene sets shown for each analysis. Gene sets significant at FDR ≤ 0.25 shown in bold

*NES* Normalized Enrichment, *Pvalue* Nominal *P* value, *FDR* False Discover Rate (Q value)

Other enriched categories of note include Wnt/B-catenin signaling, hedgehog signaling, and terms suggestive of modifications to cytokine signaling such as the inflammatory response, TNF alpha signaling, as well as both Interferon alpha and gamma responses. Genes overexpressed in the scale-biter at stages 48 hpf, 8 dpf, and 15 dpf were enriched for functions related to the estrogen response, while genes underexpressed in the durophage at 8 dpf and 15 dpf were enriched for functions related to the estrogen response.

Also of note is that we observed enrichment for pathways related to neuronal development and functioning at 48 hpf and 96 hpf as well as melanogenesis at 8 dpf, thereby highlighting both that brain tissue was included in the embryonic stage samples and that our data likely reflect species differences in addition to skull morphology such as behavior and pigmentation (Additional file 9: Table S6 and Additional file 10: Table S7).

### Identification of a set of genes that may contribute to jaw morphological variation in pupfishes

**Fig. 4:**
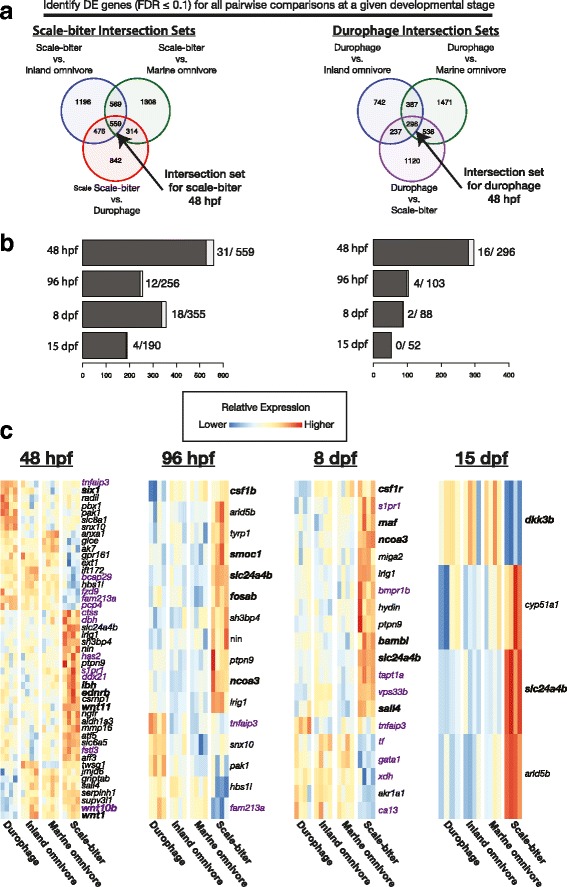
Differentially expressed (DE) genes for four developmental stages of cranial development in four pupfish taxa. These DE genes include genes which may be contributing to jaw morphological differences among taxa. **a** Genes associated with the development of distinctive skull morphologies were selected as the intersection of genes differentially expressed in all pairwise comparisons at a given stage to either the scale-biter or durophage, the two species with extreme morphologies. **b** Histograms show number of genes in each intersection. White portion of bars correspond to the number of genes in each set that are annotated to affect bone. Numbers to right of bars give number of genes annotated to affect bone and total numbers of genes in each intersection set. **c** Relative expression of genes annotated to affect skull bones (*black*) or simply bone (*purple*) at each stage in our dataset highlight the relative over- and underexpression of *wnt* ligands in the scale-biter and durophage respectively at 48 hpf, and that a number of genes are differentially expressed at multiple stages. Genes in bold are highlighted in the text

To identify genes in our dataset that might be contributing to skull morphological variation, we found the intersection set of genes at each stage that were differentially expressed (DE; FDR ≤ 0.1 and log2 fold change ≥ 0.2) in all three possible comparisons to either the scale-biter or durophage, the two morphologically extreme species (Fig. 4a; Additional file 11: Figure S4)*.* In our study, genes called DE by edgeR in any pairwise comparison were typically differentially expressed by between 1.2 fold to 1.5 fold at an FDR cutoff of 0.1 (median ranged from 1.3–1.8 fold difference across all comparisons; Additional file 12: Figure S5, Additional file 13: Figure S6, Additional file 14: Figure S7 and Additional file 15: Figure S8). Selecting genes by a more stringent 1.5-fold or 2-fold change does not affect our major conclusions, though several genes would not be identified (see discussion). Intersection sets identified in this way include ~50–600 genes that are over or underexpressed in either the scale-biter or durophage species at a particular stage (Fig. 4b). Below, we refer to these as intersection sets.

Differentially expressed genes in the intersection sets were typically either over or underexpressed in just one taxon relative to the other three (Fig. 4c; Additional file 16: Figure S9). Only between 5 and 18 genes were found to be DE in both the scale-biter and durophage sets at a given stage. For example, *bbs12* was overexpressed in the scale-biter and underexpressed in the durophage at 48 hpf. This contrasts to a hypothesized scenario where most differentially expressed genes are alternately up or down regulated in the scale-biter and durophage, with the two omnivore populations being intermediate. Further investigation of differentially expressed genes in the scale-biter and durophage confirm that different sets of DE genes characterize these extreme phenotypes relative to the omnivores.

Genes in the intersection sets have varied functional roles: growth factor signaling molecules, cell cycle regulators, apoptosis related molecules, extracellular matrix molecules, solute carriers, cytokine/chemokine molecules, and transcription factors known to be involved in bone development and remodeling (Tables 4, 5, 6 and 7; Additional file 17: Table S8, Additional file 18: Table S9, Additional file 19: Table S10, Additional file 20: Table S11, Additional file 21: Table S12, Additional file 22: Table S13, Additional file 23: Table S14 and Additional file 24: Table S15). We find a number of molecules which function in metabolism, fatty acid synthesis and lipid transport, and protein sorting. We find members of five growth factor/paracrine signaling pathways that play roles in bone growth and remodeling including multiple Wnt ligands and the Wnt receptor *fzd9*, Igf binding proteins *igfbp2* and *igfbp5*-like, Bmp receptor *bmpr1b* among other Tgf-*β* related molecules, hedgehog antagonist *hhip1*, and a number of cytokine/chemokine ligands and receptors (Tables 4, 5, 6 and 7; Additional file 17: Table S8, Additional file 18: Table S9, Additional file 19: Table S10, Additional file 20: Table S11, Additional file 21: Table S12, Additional file 22: Table S13, Additional file 23: Table S14 and Additional file 24: Table S15).

**Table 4:** Select genes from all four intersection sets that are overexpressed in the scale-biter

Gene Group	Gene Name	Intersection Set			
Apoptosis					
*aifm3*	apoptosis inducing factor, mitochondria associated 3	48hpf		8dpf	
*aven*	apoptosis, caspase activation inhibitor	48hpf	96hpf		
*bnip3*	BCL2/adenovirus E1B 19 kDa interacting protein 3	48hpf			
LOC107103375	caspase recruitment domain-containing protein 8-like		96hpf		
Bardet–Biedl syndrome					
*bbs12*	Bardet-Biedl syndrome 12	48hpf			
Calcium Signaling					
*anxa11*	annexin A11	48hpf	96hpf		
*calb2*	calbindin 2	48hpf			
*calcrl*	calcitonin receptor like receptor	48hpf			
LOC107082646	calpain-1 catalytic subunit-like	48hpf			
LOC107081484	calpain-1 catalytic subunit-like	48hpf			
LOC107102261 (*capn1a*)	calpain-1 catalytic subunit-like	48hpf			
LOC107092503	calpain-9-like	48hpf	96hpf	8dpf	
LOC107099392	calpain-9-like		96hpf		
*clstn3*	calsyntenin 3	48hpf			
LOC107084037 (*s100 s*)	protein S100-A1-like	48hpf	96hpf		
Cell Adhesion					
*ncam1*	neural cell adhesion molecule 1	48hpf			
LOC107088148	cadherin-like protein 26			8dpf	
LOC107084761	claudin-9-like			8dpf	
Cell Cycle					
*bora*	bora, aurora kinase A activator	48hpf	96hpf		
Cytokine/Chemokine					
LOC107100215 (*cxcr3.2*)	C-X-C chemokine receptor type 3-like	48hpf			
LOC107100210	C-X-C chemokine receptor type 4-B-like	48hpf			
LOC107091150 (*il12rb2l*)	interleukin-12 receptor subunit beta-2-like	48hpf	96hpf		
*il4r*	interleukin 4 receptor				15dpf
LOC107087156 (*il2rb*)	interleukin-2 receptor subunit beta-like				15dpf
LOC107092801 (*ngfr*)	tumor necrosis factor receptor superfamily member 16-like	48hpf			
*tnfrsf21*	tumor necrosis factor receptor superfamily member 21	48hpf	96hpf		
*clcf1*	cardiotrophin-like cytokine factor 1			8dpf	
Chaperone/Heat Shock					
LOC107090055 (*dnaja3b*)	dnaJ homolog subfamily A member 3, mitochondrial-like		96hpf		
LOC107081529	heat shock 70 kDa protein 12A–like				15dpf
Lipid Transport					
LOC107095514	apolipoprotein A-IV-like	48hpf			
*apof*	apolipoprotein F	48hpf			
Growth Factor					
LOC107086851 (*ednrba*)	endothelin B receptor-like	48hpf			
LOC107083579 (*epha6*)	ephrin type-A receptor 6-like	48hpf			
LOC107084161	fibroblast growth factor 13-like	48hpf			
*flt1*	fms-related tyrosine kinase 1	48hpf			
BMP					
*bambi*	BMP and activin membrane-bound inhibitor			8dpf	
*bmpr1b*	bone morphogenetic protein receptor type IB			8dpf	
IFG signaling					
*igfbp2*	insulin like growth factor binding protein 2	48hpf	96hpf	8dpf	15dpf
LOC107084241	insulin-like growth factor-binding protein 5	48hpf	96hpf	8dpf	15dpf
*igflr1*	IGF like family receptor 1			8dpf	
Matrix					
*adam22*	ADAM metallopeptidase domain 22	48hpf			
*crtap*	cartilage associated protein	48hpf	96hpf		
LOC107101759	collagen alpha-1(XXVIII) chain-like	48hpf			
*col5a3*	collagen, type V, alpha 3	48hpf			
*col16a1*	collagen, type XVI, alpha 1	48hpf			
LOC107098087 (*col8a1b*)	collagen alpha-1(VIII) chain-like		96hpf		
LOC107101792 (*col15a1b*)	collagen alpha-1(XV) chain-like		96hpf		
LOC107084752	integrin beta-3-like	48hpf			
*fstl3*	follistatin-like 3 (secreted glycoprotein)	48hpf			
LOC107092333 (*fndc7*)	fibronectin type III domain-containing protein 7-like	48hpf			
LOC107081663 (*mmp16b*)	matrix metalloproteinase-16-like	48hpf			
*otol1*	otolin 1	48hpf			
LOC107089461 (*phlda2*)	pleckstrin homology-like domain family A member 2	48hpf			
*plekhh2*	pleckstrin homology, MyTH4 and FERM domain containing H2	48hpf	96hpf	8dpf	15dpf
*plod1*	procollagen-lysine, 2-oxoglutarate 5-dioxygenase 1	48hpf			
LOC107097692 (*sparcl1*)	SPARC-like protein 1	48hpf			
*smoc1*	SPARC related modular calcium binding 1		96hpf		
*fstl4*	follistatin-like 4			8dpf	
LOC107101284	thrombospondin type-1 domain-containing protein 7A–like			8dpf	
LOC107101285	thrombospondin type-1 domain-containing protein 7A–like			8dpf	
Muscle					
LOC107095331	myosin-16-like	48hpf			
LOC107082773 (*tpm1*)	tropomyosin alpha-1 chain-like	48hpf			
LOC107089161	troponin I, slow skeletal muscle-like	48hpf	96hpf		
Tgf-beta					
*tgfbi*	transforming growth factor beta induced	48hpf			
Transciption Factor					
*evx1*	even-skipped homeobox 1	48hpf			
*meox1*	mesenchyme homeobox 1	48hpf			
*sp4*	Sp4 transcription factor	48hpf	96hpf	8dpf	
LOC107087726	sal-like protein 1		96hpf		
LOC107093901 (*ncoa3*)	nuclear receptor coactivator 3-like		96hpf	8dpf	
*sall4*	spalt-like transcription factor 4			8dpf	
LOC107084340 (*mafb*)	transcription factor Maf-like			8dpf	
Wnt					
*wnt11*	wingless-type MMTV integration site family member 11	48hpf			
*ilkap*	ILK associated serine/threonine phosphatase		96hpf		
Other					
*lbh*	limb bud and heart development	48hpf			
LOC107088691 (*npdc1a*)	neural proliferation differentiation and control protein 1-like	48hpf			
*rps6kl1*	ribosomal protein S6 kinase like 1	48hpf	96hpf		
LOC107087452	ribosomal protein S6 kinase beta-1-like			8dpf	15dpf
LOC107095875	sex comb on midleg-like protein 4	48hpf			
LOC107102698	tissue factor-like	48hpf			
*slc24a4*	solute carrier family 24 (sodium/potassium/calcium exchanger), member 4	48hpf	96hpf	8dpf	
*acp7*	acid phosphatase 7, tartrate resistant (putative)			8dpf	
*vwa1*	von Willebrand factor A domain containing 1			8dpf	
*vwde*	von Willebrand factor D and EGF domains			8dpf	
LOC107081298	von Willebrand factor-like			8dpf	
*c1galt1*	core 1 synthase, glycoprotein-N-acetylgalactosamine 3-beta-galactosyltransferase 1				15dpf
*mpeg1*	macrophage expressed 1				15dpf

Shown are pupfish gene names with zebrafish gene names in parentheses

**Table 5:** Select genes from all four intersection sets that are underexpressed in the scale-biter

Gene Group	Gene Name	Intersection Set			
Apoptosis					
*api5*	apoptosis inhibitor 5	48hpf			
LOC107081897	caspase-1-like				15dpf
Bardet–Biedl syndrome					
*bbs2*	Bardet-Biedl syndrome 2	48hpf	96hpf		
*bbs5*	Bardet-Biedl syndrome 5	48hpf			
Calcium Signaling					
LOC107088690	annexin A3-like	48hpf			
*capn5*	calpain 5	48hpf			
*calu*	calumenin	48hpf			
Cell Adhesion					
*cdh20*	cadherin 20, type 2	48hpf			
*cdh17*	cadherin 17, LI cadherin (liver-intestine)			8dpf	
*cldn12*	claudin 12	48hpf			
LOC107103695	claudin-3-like	48hpf			
LOC107103700	claudin-4-like	48hpf			
LOC107084763	claudin-4-like			8dpf	
LOC107100707	sialoadhesin-like				15dpf
Cell Cycle					
*cdca7*	cell division cycle associated 7	48hpf			
*ccny*	cyclin Y	48hpf	96hpf	8dpf	
Cytokine/Chemokine					
*il6st*	interleukin 6 signal transducer	48hpf	96hpf		15dpf
LOC107089554	interleukin-21 receptor-like			8dpf	
LOC107096536	C-C motif chemokine 3-like			8dpf	
Chaperone/Heat Shock					
LOC107082103	dnaJ homolog subfamily B member 9-like	48hpf	96hpf	8dpf	15dpf
LOC107091513	dnaJ homolog subfamily C member 16-like	48hpf		8dpf	
LOC107095621	dnaJ homolog subfamily C member 3-like	48hpf			
LOC107088964	dnaJ homolog subfamily B member 5-like		96hpf		
Lipid Transport					
*fabp6*	fatty acid binding protein 6, ileal	48hpf			
LOC107101094	fatty acid-binding protein 10-A, liver basic-like	48hpf			
*fabp3*	fatty acid binding protein 3, muscle and heart		96hpf	8dpf	
LOC107096181	fatty acid-binding protein, brain-like		96hpf	8dpf	
LOC107081410	fatty acid-binding protein, heart-like		96hpf		15dpf
LOC107086290	fatty acid-binding protein, liver-type-like			8dpf	
LOC107082973	apolipoprotein A-IV-like			8dpf	
LOC107095516	apolipoprotein Eb-like			8dpf	
Growth Factor					
*sh2d3c*	SH2 domain containing 3C			8dpf	
BMP					
LOC107089450	activin receptor type-2B-like	48hpf	96hpf		
Matrix					
LOC107082466	cartilage acidic protein 1-like	48hpf	96hpf		15dpf
LOC107093562	collagen alpha-1(XXVIII) chain-like	48hpf			
LOC107094934	collagen alpha-2(I) chain-like		96hpf		
LOC107086858	collagen alpha-4(IV) chain-like			8dpf	
LOC107097307	disintegrin and metalloproteinase domain-containing protein 10-like	48hpf			
*fsd1*	fibronectin type III and SPRY domain containing 1	48hpf			
*fbln2*	fibulin 2	48hpf	96hpf	8dpf	15dpf
*plekhg1*	pleckstrin homology and RhoGEF domain containing G1	48hpf			
LOC107097201	integrin alpha-D-like		96hpf		
Muscle					
*myo7a*	myosin VIIA	48hpf			
LOC107097407	myosin-7B-like	48hpf	96hpf		
LOC107087696	myosin-11-like			8dpf	
Tgf-beta					
*tbrg4*	transforming growth factor beta regulator 4	48hpf			
Transciption Factor					
*irx6*	iroquois homeobox 6	48hpf			
*gata6*	GATA binding protein 6			8dpf	
LOC107090297	GATA zinc finger domain-containing protein 14-like				15dpf
Wnt					
*fzd9*	frizzled class receptor 9	48hpf			
*wnt2b*	wingless-type MMTV integration site family member 2B	48hpf			
LOC107090002	dixin-A-like		96hpf	8dpf	
LOC107098026	dickkopf-related protein 3-like				15dpf
Other					
LOC107101384	adipocyte plasma membrane-associated protein-like	48hpf			
*clptm1*	cleft lip and palate associated transmembrane protein 1	48hpf			
*fkbp2*	FK506 binding protein 2	48hpf			
*foxj3*	forkhead box J3	48hpf			
*oraov1*	oral cancer overexpressed 1	48hpf		8dpf	15dpf
*smarcd2*	SWI/SNF related, matrix associated, actin dependent regulator of chromatin, subfamily d, member 2	48hpf			
LOC107105123	syndecan-2-like	48hpf			
LOC107087936	transcription regulator protein BACH1-like		96hpf		
LOC107083667	alkaline phosphatase-like			8dpf	
*setd6*	SET domain containing 6	48hpf	96hpf	8dpf	15dpf
*ptgr2*	prostaglandin reductase 2				15dpf
LOC107094401	kallikrein-7-like				15dpf

Shown are pupfish gene names with zebrafish gene names in parentheses

**Table 6:** Select genes from all four intersection sets that are overexpressed in the durophage

Gene Group	Gene Name	Intersection Set			
Apoptosis					
LOC107095016 (*aifm4*)	apoptosis-inducing factor 3-like	48hpf	96hpf	8dpf	15dpf
LOC107104525	apoptosis-stimulating of p53 protein 2-like			8dpf	
*bag1*	BCL2 associated athanogene 1	48hpf			
LOC107103519	caspase recruitment domain-containing protein 8-like				15dpf
Calcium Signaling					
*anxa1*	annexin A1	48hpf			
*capn3*	calpain 3	48hpf			
*capn5*	calpain 5	48hpf			
Cell Adhesion					
LOC107095872 (*cdh4*)	cadherin-4-like	48hpf			
LOC107093297	claudin-4-like	96hpf			
Cytokine/Chemokine					
*tnfaip3*	TNF alpha induced protein 3	48hpf	96hpf	8dpf	
Chaperone/Heat Shock					
*dnajc27*	Hsp40	48hpf		8dpf	
LOC107099519 (*dnajc11*)	dnaJ homolog subfamily C member 11-like	48hpf			
Growth Factor					
LOC107091872 (*epha4b*)	ephrin type-A receptor 3-like	48hpf			
LOC107090696	ephrin type-B receptor 2-like	48hpf			
LOC107086153	platelet-derived growth factor receptor-like protein			8dpf	
Extracellular Matrix					
LOC107102808	integrin beta-2-like	48hpf			
LOC107088257	integumentary mucin A.1-like	48hpf			
*col16a1*	collagen, type XVI, alpha 1			8dpf	
*thsd1*	thrombospondin type 1 domain containing 1		96hpf		
LOC107083667	alkaline phosphatase-like				15dpf
Muscle					
*myoz3*	myozenin3				15dpf
Transcription Factors					
*atoh8*	atonal bHLH transcription factor 8	48hpf			
*gatad2b*	GATA zinc finger domain containing 2B	48hpf			
*ncoa1*	nuclear receptor coactivator 1	48hpf	96hpf	8dpf	
LOC107081391	nuclear receptor coactivator 1-like	48hpf	96hpf	8dpf	15dpf
*six1*	SIX homeobox 1	48hpf			
*six4*	SIX homeobox 4	48hpf			
Other					
LOC107098473	von Willebrand factor A domain-containing protein 7-like	48hpf			
LOC107084756	toll-like receptor 2 type-2				15dpf

Shown are pupfish gene names with zebrafish gene names in parentheses

**Table 7:** Select genes from all four intersection sets that are underexpressed in the durophage

Gene Group	Gene Name	Intersection Set			
Apoptosis					
*bag2*	BCL2 associated athanogene 2	48 hpf			
LOC107082055 (*casp8l2*)	caspase-8-like	48 hpf			
Bardet–Biedl syndrome					
*bbs12*	Bardet-Biedl syndrome 12	48 hpf	96 hpf		
Calcium Signaling					
*anxa10*	annexin A10	48 hpf			
LOC107089898 (*camkk1a*)	calcium/calmodulin-dependent protein kinase kinase 1-like		96 hpf		15 dpf
Cell Adhesion					
*cd302*	CD302 molecule	48 hpf	96 hpf		
LOC107104323 (*bub1*)	mitotic checkpoint serine/threonine-protein kinase BUB1-like	48 hpf			
LOC107097627	protocadherin beta-16-like		96 hpf		
LOC107100986 (*pcdh10b*)	protocadherin-10-like		96 hpf		
LOC107105174	cell adhesion molecule 2-like			8dpf	
Cell Cycle					
LOC107092486 (*aunip*)	aurora kinase A and ninein-interacting protein-like	48 hpf			
*cdc20*	cell division cycle 20	48 hpf			
*cdca7l*	cell division cycle associated 7-like	48 hpf	96 hpf		
Cytokine/Chemokine					
LOC107085925	C-C motif chemokine 25-like	48 hpf			
*traf4*	TNF receptor associated factor 4		96 hpf		
LOC107095001 (*csf1ra*)	macrophage colony-stimulating factor 1 receptor 1-like			8dpf	
LOC107090375 (csf1b*)*	uncharacterized (macrophage colony stimulating factor 1b)		96 hpf		
Chaperone/Heat Shock					
LOC107086201	heat shock 70 kDa protein 12A–like	48 hpf			
Growth Factor					
LOC107082400	endothelin B receptor-like	48 hpf			
LOC107086153	platelet-derived growth factor receptor-like protein	48 hpf			
*flt1*	fms-related tyrosine kinase 1		96 hpf		
IFG signaling					
LOC107082691 (*igfbp7*)	insulin-like growth factor-binding protein 7		96 hpf		
Matrix					
LOC107101414	FRAS1-related extracellular matrix protein 2-like	48 hpf			
LOC107103703	FRAS1-related extracellular matrix protein 2-like	48 hpf			
LOC107094308	integrin beta-1-like	48 hpf			
*pcolce2*	procollagen C-endopeptidase enhancer 2	48 hpf			
*otol1*	otolin 1		96 hpf	8dpf	
Muscle					
LOC107103762 (*mybpha*)	myosin-binding protein H-like			8dpf	15 dpf
BMP/Tgf-beta					
*twsg1*	twisted gastrulation BMP signaling modulator 1	48 hpf			
Transciption Factors					
*dbx2*	developing brain homeobox 2	48 hpf			
*hes3*	hes family bHLH transcription factor 3	48 hpf			
*sall4*	spalt-like transcription factor 4	48 hpf			
Wnt					
LOC107099028 (*tmem88b*)	transmembrane protein 88-like	48 hpf			
*wnt1*	wingless-type MMTV integration site family member 1	48 hpf			
*wnt10b*	wingless-type MMTV integration site family member 10b	48 hpf			
Other					
*sipa1l3*	signal-induced proliferation-associated 1 like 3	48 hpf		8dpf	
*tfpi2*	tissue factor pathway inhibitor 2	48 hpf			
LOC107100889	myeloid-associated differentiation marker homolog			8dpf	
LOC107104594	TRPM8 channel-associated factor homolog			8dpf	
*mos*	v-mos Moloney murine sarcoma viral oncogene homolog				15 dpf

Shown are pupfish gene names with zebrafish gene names in parentheses

Overrepresentation analysis indicated that intersection sets were generally not significantly enriched for GO terms at an FDR threshold of ≤0.1 (Additional file 25: Table S16, Additional file 26: Table S17, Additional file 27: Table S18, Additional file 28: Table S19, Additional file 29: Table S20 and Additional file 30: Table S21). Notable exceptions were that the intersection set for the scale-biter at 48 hpf was significantly enriched for genes related to cilium and plasma membrane, and the intersection set for the scale-biter at 8 dpf was enriched for carboxylic acid and oxoacid metabolic processes.

To further investigate whether genes known to affect bony skull elements are within the intersection sets, we curated a list of over 1700 genes from databases and from literature searches. We downloaded lists of genes that have known craniofacial phenotypes from the Zebrafish Model Organism Database (ZFIN), the Mouse Genome Informatics Database (MGI), and NCBI Phenotype-Genotype Integrator (PheGenI) using the search terms “Cranial Cartilage”, “Cranium”, “Pharyngeal Arch Cartilage”, “Ventral Mandibular Arch”, “Craniofacial Development” (ZFIN), “Jaw”, “Maxilla”, “Skull”, “Craniofacial” (MGI), and “Face”, “Jaw Abnormalities”, “Cleft Lip”, “Cleft Palate” (PheGenI). We also downloaded genes annotated by the Gene Ontology Consortium with functions related to craniofacial morphology or bone.

More than 95% of the genes discovered through intersection sets have not been previously annotated with functions directly related to craniofacial morphology or bone (Fig. 4b,c). Of the genes in the intersection sets that are annotated to affect bone, most are also annotated to have craniofacial phenotypes indicating that our literature and database searches were likely to have been fairly comprehensive (Fig. 4b,c). To assess whether we identified a greater number of annotated genes in the intersection sets than would be expected by chance, we calculated a probability distribution by identifying the number of curated genes in 1000 randomly drawn sets of equal size to each of the intersection sets. We found that none of the sets contained significantly more genes with previously researched craniofacial phenotypes than would be expected by chance alone suggesting that the intersection sets are not statistically enriched for genes already known, largely from biomedical research, to affect skull morphology.

The lack of statistically detected enrichment does not necessarily eliminate curated genes as being important. For example, if morphological differences are produced by modified expression of only a few genes then this would not be detected by overrepresentation analyses. Thus, we also consider potentially relevant annotated genes found to be DE in our dataset (Fig. 4c). We find multiple Wnt ligands either overexpressed in the scale-biter (*wnt11*), or underexpressed in in the durophage (*wnt1*, *wnt10b*) at 48 hpf. Wnt signaling is well known to affect craniofacial morphology [25–27, 29, 51]. While we note that Wnt ligands were typically DE by less than 1.5 fold, these data along with GSEA results (Tables 1, 2 and 3; Additional file 5: Table S2, Additional file 6: Table S3, Additional file 7: Table S4, Additional file 8: Table S5, Additional file 9: Table S6 and Additional file 10: Table S7), suggest that Wnt signaling may be differentially activated in the two species with extreme jaw morphologies at an early stage of development.

A number of transcription factors are within the intersection sets, including *six1*, *twsg1*, *sall4* at 48 hpf, *fosab, ncoa3* at 96 hpf, and *gata1, sall4, ncoa3, and maf* at 8 dpf (Fig. 4c). We found cytokine/chemokine signaling molecules with known craniofacial phenotypes including a putative macrophage colony stimulating factor 1 (*csf1b*) at 96 hpf, and its receptor *csf1r* at 8 dpf. Both *csf1b* and *csf1r*, are known to play important roles in osteoclast differentiation [52–54]. Other genes of note include *slc24a4* (associated with enamel formation in human) [55], *smoc1* (associated with craniofacial morphology in human) [56, 57], *lbh* (craniofacial evolution in cichlids) [58], *ednrb* (endothelin signaling) [59, 60], *bambi* (Bmp signaling and differential expression during bone remodeling) [61], and *dkk3b* (craniofacial evolution in finches) [62].

### Constitutive differential expression of genes in the scale-biter and durophage taxa

Gene expression is dynamic, and it is possible that genes will be differentially expressed at only critical periods of time during development. Alternatively, many genes are constitutively expressed and may be differentially expressed over relatively long blocks of developmental time. At a transcriptomic level different genes could follow either of these patterns. We asked what percentage of genes in the intersection sets follow a pattern of being differentially expressed at a single time point versus what percentage are differentially expressed over more than one time point.

**Fig. 5:**
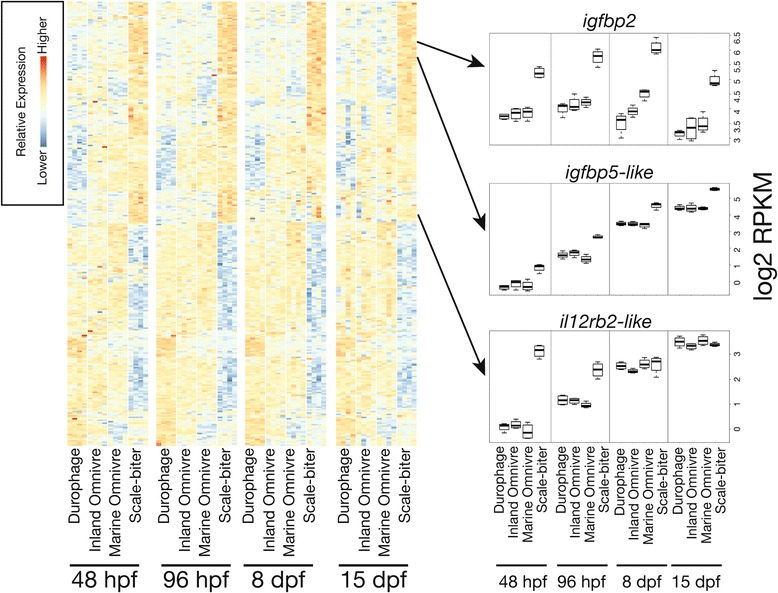
Relative expression of differentially expressed genes shows that many genes are differentially expressed in all four stages. Heatmap shows relative expression at each stage for genes represented in two or more durophage or scale-biter intersection sets. The overall pattern of relative expression trends to be similar at all four stages for this set of genes indicating constitutive differential expression. For instance, genes over- or underexpressed in the scale-biter tend to also be over- or underexpressed at other stages as well. Boxplots show expression patterns for three representative genes that may also be relevant to skull morphology based on function. Note how the pattern of expression for *igfbp2*, *igfbp5* is similar across all four stages even when the species-wide mean expression changes across stages, while the interleukin 12 receptor paralog, *il12rb2-like*, is greatly overexpressed in the scale-biter at embryonic stages, but not at post-hatching stages

Of the genes in all four scale-biter intersection sets, 227 (22.0%) occur in an intersection set at two or more stages. Similarly, 79 (18.5%) of all durophage genes occur in an intersection set at two or more stages. In fact, most of these genes may be differentially expressed over the entire course of developmental stages sampled if we relax the criterion of statistical significance (Fig. 5). Genes that were overexpressed or underexpressed at one stage tended to be overexpressed or underexpressed respectively at all other stages as well, even if not sufficiently so to be deemed statistically significant at an FDR ≤ 0.1. However, we recognize that there can be important functional impact of even slight differential expression [63]. Therefore, when differential expression is in the same direction (over- or underexpressed) in all four developmental stages sampled, we refer to these genes as constitutively differentially expressed.

This pattern holds even when the level at which a gene is expressed changes through development as seen in the representative gene boxplots (e.g. *igfbp5-like* in Fig. 5). Other genes, however, did not follow this pattern throughout the experiment. For instance, an interleukin 12b receptor paralog is expressed in the scale-biter at levels typical for larval pupfish of all 4 taxa, but this gene is not differentially expressed at post-hatching stages (Fig. 5). These results suggest that while ~80% of genes in our intersection sets are differentially expressed at only a single time point during development, approximately 20% of the genes in our scale-biter and durophage intersection sets are constitutively differentially expressed during the period of development we sampled. This may be an underestimate of constitutive expression and we suspect that a much greater proportion of genes are constitutively differentially expressed (see discussion).

### Expression of previously identified candidate genes in pupfishes

A fundamental question in evolutionary biology concerns the extent to which the genetic sources of phenotypic diversity are shared across taxa. Early work identified modifications to *Bmp4* and calmodulin associated with jaw diversification in both African cichlids and Galapagos finches [20, 21, 64], as well as modification to *Bmp4* contributing to beak differences in ducks and chickens [65]. Given the great divergence time between cichlids and finches, these studies raised the possibility that modifications to the expression of *Bmp4* and calmodulin might be responsible for jaw diversity in other taxa, or at least across all vertebrates.

We asked whether candidate genes known to produce jaw diversity in other vertebrates were also differentially expressed at any stage among distinct jaw phenotypes of pupfishes. We also explored whether genes affecting cranial cartilages identified from a zebrafish mutagenesis screen were differentially expressed among species of pupfishes with different jaw phenotypes [66, 67]. In contrast to the intersection sets, here we are interested in genes that may be differentially expressed in even a single pairwise comparison.

**Fig. 6:**
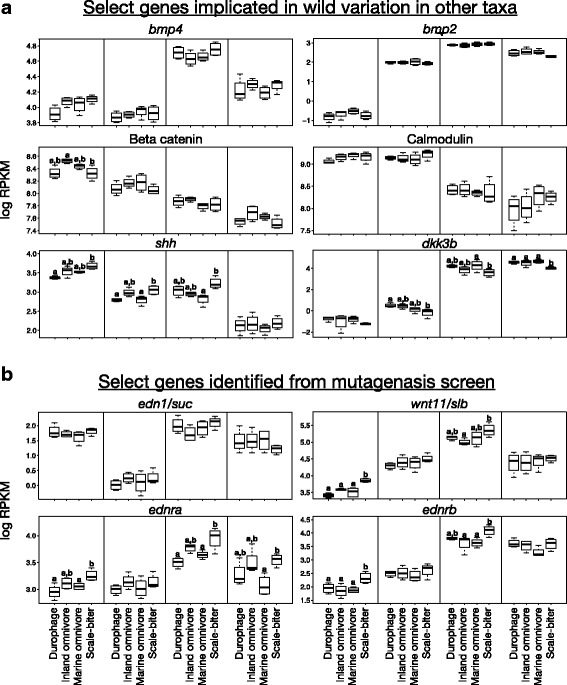
Expression of several candidate genes associated with jaw morphological diversification in other taxa (**a**) or identified as affecting jaw and cranial morphology from a zebrafish mutagenesis screen (**b**). **a** Boxplots of gene expression levels for each species at all four stages of development. Gene expression values were measured as log2 transformed reads per kilobase per million reads (RPKM) for a set of candidate genes known to affect skull or cranial morphology in other wild vertebrates. These genes are not differentially expressed among different species of pupfish, with the exception of *shh* that tends to be slightly overexpressed in the scale-biter, and *dkk3b* which is underexpressed in the scale-biter. **b** Expression of four genes identified from a zebrafish mutagenesis screen that may contribute to jaw morphological differences among species of pupfish. Note that while *edn1* is not differentially expressed, two endothelin receptors are. Letters indicate significant differential expression (FDR ≤ 0.1) in pairwise comparisons at each stage. Letters are omitted from stages where no pairwise comparisons are significant

Expression levels of *bmp4*, *bmp2*, calmodulin, *ptch1*, *β*-catenin and *tgfr2*, genes associated with changes to jaw shape (*bmp4*, calmodulin, *tgfr2*, *ptch1*) or size (*bmp2*) in finches or cichlids, are expressed at similar levels among pupfishes at all four stages (Fig. 6a; data for some genes not plotted). In contrast, however, there are several genes differentially expressed among pupfishes as well as cichlids and finches. Paralogs to *camkII* and other calmodulin dependent kinases are differentially expressed among pupfish taxa (Additional file 31: Table S22), and *shh* tends to be slightly overexpressed in the scale-biter (Fig. 6a). The Wnt signaling antagonist *dkk3b*, but not *dkk3a*, is underexpressed in the scale-biter at 15 dpf. The transcriptional activator, *lbh*, is overexpressed in the scale-biter at 48 hpf (Fig. 4) [58].

Three candidate genes emerging from a zebrafish mutagenesis screen, *wnt11*, *ednra*, and *ednrb* [59, 66, 67], are overexpressed in the scale-biter, and may contribute to the extreme jaw morphology of this species. Wnt11 function is absent or reduced in zebrafish *Silberblick* (*wnt11*/*slb*) mutants that exhibit dramatic abnormalities to the anterior portions of the skull [23, 51, 66] reminiscent of the differences in anterior skull bone morphology between the scale-biters and other species of pupfishes (e.g. oral jaw bone length). We also find that *ednra* and *ednrb*, receptors for *Edn1* signaling, are differentially expressed across species of pupfishes (Fig. 6b). *Edn1* was identified as the zebrafish *Sucker* (*edn1*/*suc*) mutant, and work in mouse and chicken has indicated that disrupting the expression of *Ednra* also results in abnormal jaw morphologies [59, 68–70].

### Expression in pupfishes of osteoblast and osteoclast marker genes

Genes found to be differentially expressed among pupfishes contained a number of molecules known to affect osteoblast and osteoclast differentiation, proliferation, and apoptosis. We were thus interested in whether genes commonly used as genetic markers of osteoblast and osteoclast activity were differentially expressed. We investigated more closely the expression patterns of four osteoblast marker genes *runx2*, *rankl*, *csf1b*, and alkaline phosphatase, as well as six osteoclast expressing genes including *rank*, calcitonin receptors *calcrl* and *calcr*, cathepsin K, and *acp5* (tartrate resistant acid phosphatase) in order to determine whether there was a signal of cell types being more or less active or abundant in some species relative to others.

**Fig. 7:**
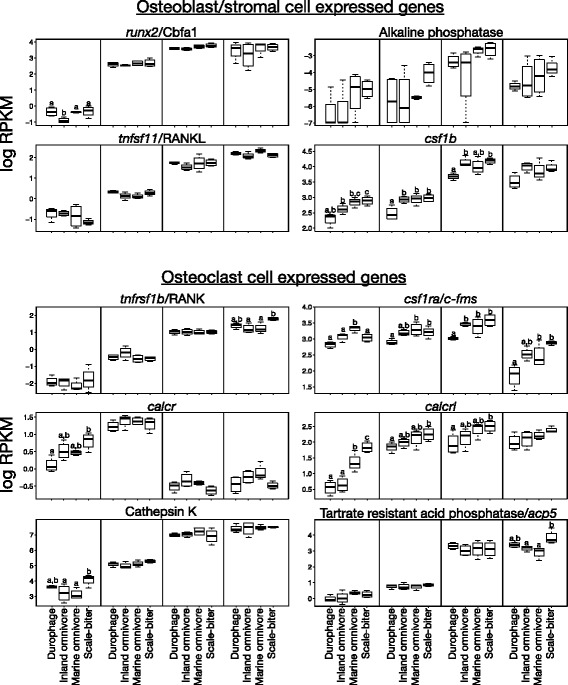
Expression of genes commonly used as molecular markers of osteoblast and osteoclast cells indicate that genes functioning in osteoblast differentiation are typically not differentially expressed among species of pupfish, while a number of osteoclast expressed genes tend to be overexpressed in the scale-biter and underexpressed in the durophage. Gene expression values were measured as log2 transformed RPKM similar to Fig. 6. Letters indicate significant differential expression (FDR ≤ 0.1) in pairwise comparisons at each stage. Letters are omitted from stages where no pairwise comparisons are significant

Genes associated with osteoblast activity were typically not differentially expressed among species of pupfishes (Fig. 7). The one exception was a putative ortholog of the zebrafish macrophage colony stimulating factor 1b (*csf1b*), a gene also identified in the intersection sets, that tended to be constitutively underexpressed in the durophage at all developmental stages. Mammalian osteoblast/stromal cells are known to express *Csf1* as a molecule that affects osteoclast differentiation, recruitment, and activity [52–54].

A number of osteoclast expressing genes were differentially expressed in our dataset. Calcitonin receptors and cathepsin K were slightly overexpressed in the scale-biter at embryonic stages of development, and both *rank* and *acp5* were overexpressed in the scale-biter at 15 dpf, during a period of larval growth. These data suggest that osteoclast activity or number may differ among species of pupfishes, with perhaps osteoclast activity lower in the durophage and relatively higher in the scale-biter. Intriguingly, osteoclast marker genes were not differentially expressed in all pairwise comparisons and genes that were differentially expressed tended to be DE at different stages. This perhaps suggests that either only osteoclast activity, and not number, is differentially activated, or that RNA-seq is not sensitive enough to pick up differential expression in each of these genes.

## Discussion

This paper presents foundational data as a first step towards addressing the fundamental question of how phenotypic variation is produced during the early stages of diversification, here among three closely related species. We use RNA-seq to study gene expression divergence associated with ecological and morphological diversification in three species of *Cyprinodon* pupfishes that are estimated to have diverged in only the last 6000–10,000 years [12, 35]. RNA-seq reveals the amount of mRNA transcription, and may detect both changes to spatial location of genes as well as altered onset or offset of expression across species.

Gene expression divergence is known to accumulate with time since common ancestry [71–73]. Even at the shallow divergence time studied here, patterns of gene expression divergence appear to reflect phylogenetic estimates of time since common ancestry, at least partially. Our data support other studies placing the marine omnivore population as the sister group to a clade of San Salvador pupfishes [31, 39], suggesting that the durophage and scale-biter species evolved from one or multiple inland omnivore population(s) present in the salt water lakes of San Salvador Island. We find that the marine omnivore differs from the inland taxa by a single PC axis at each stage, possibly reflecting the divergence of the inland forms following colonization of San Salvador by the marine lineage. Interestingly, in contrast to an expected scenario where morphologically similar omnivore populations are also most similar in gene expression patterns, principal component analyses of gene expression variance grouped the inland omnivore with the endemic inland durophage rather than the morphologically similar marine omnivore. This unexpected result could reflect time since common ancestry or ongoing introgression among these two taxa [31, 39] or both.

Gene set enrichment analysis suggested that a number of conserved cellular processes may be differentially regulated among species of pupfishes including Wnt signaling, hedgehog signaling, myogenesis, adipogenesis, the inflammation response, and fatty acid metabolism. Many of the gene sets identified as enriched are related to cell cycle regulation, perhaps indicating differences in rates of cell proliferation among species. Of particular note was enrichment for targets of Myc transcription factor activity. Myc is an immediate early response transcription factor that among other roles mediates a cellular response to growth factors. Previous work on transcriptional responses to diet in cichlid fishes and bone loading in mouse have both implicated gene expression modifications to immediate early response genes [61, 74].

GSEA suggested that the expression of genes functioning in the epithelial to mesenchymal transition, as well as the estrogen response, may be differentially modified in pupfish species. Both craniofacial morphology and pigmentation differ among pupfish species, and both of these traits are derived from neural crest cells that undergo an epithelial to mesenchymal transition prior to migration. Estrogen signaling is known to affect bone and has been shown to underlie skull sexual dimorphism in *Anolis carolinensis* [75].

### Identification of genes of interest

To identify specific genes of interest, we focused on the intersection sets of genes differentially expressed in all three comparisons to either the durophage or scale-biter, the two species with extreme morphologies. The rationale for this approach is that if a set of genes is differentially expressed in all three comparisons with an extreme phenotype, these expression differences are likely to be biologically meaningful.

Genes identified by intersection sets were typically over or underexpressed in only a single taxon (Additional file 16: Figure S9). While we cannot rule out that jaw morphological differences among these species of pupfishes are produced by fine-tuning the activity of the same upstream regulators, if this were the case we would have expected to see extensive sharing of differential expression of downstream target genes. It is intriguing to consider that differences appearing as opposite ends of a morphological spectrum (e.g. short jaws vs. long jaws) may be produced by tweaking different aspects of a jaw developmental program.

Our study compares closely related wild taxa, diverged as recently as 10,000 years. As such, we would not expect large-fold differential gene expression and we are keenly aware that subtle changes in gene expression can have significant phenotypic consequences. For instance, small changes to the quantitative amount of *Shh* expression in the developing head of chickens has substantive phenotypic consequences for craniofacial morphology [9, 63]. Evolution operates by tinkering with existing genetic/developmental processes, and the striking morphological differences among species of pupfishes may be produced by slight modifications to gene expression. Despite a number of RNA-seq or microarray studies on closely related species there is still no expectation for the magnitude of biologically relevant gene expression divergence between species. An RNA-seq study of cichlid pharyngeal jaws found modest expression changes among morphs within the range of what we find among species of pupfishes [74]. Rather than arbitrarily discarding everything except the most dramatically over or underexpressed genes, we opted to use a lower threshold of differential expression but leveraged the fact that we have three comparisons for each focal taxon in order to limit our set of DE genes to those most likely to be biologically meaningful.

We applied a log2 0.2 fold change threshold to label genes as DE, in contrast to many other gene expression studies targeting larger expression differences among treatments by using a 1.5- or 2-fold change threshold to identify genes of interest. Applying this higher threshold to our data does not substantially change our conclusions (Additional file 17: Table S8, Additional file 18: Table S9, Additional file 19: Table S10, Additional file 20: Table S11, Additional file 21: Table S12, Additional file 22: Table S13, Additional file 23: Table S14 and Additional file 24: Table S15). However, using the higher threshold, *wnt* ligands would not have been identified (*wnt* ligands were DE by 1.2–1.48 fold in any pairwise comparison). Additionally, a number of genes in the intersection sets would be excluded with a higher threshold simply because a single comparison was slightly below the threshold, although even at the higher threshold, the two other comparisons categorized them as DE. Requiring genes to be DE in all three comparisons, even with a lower threshold, is already a stringent criterion. A threshold of 1.5 or 2 fold is arbitrary, but detecting a difference in all three comparisons suggests biological meaning.

While the main focus of our project was to identify genes differentially expressed among species of pupfishes, our data are also interesting for those genes not differentially expressed. Genes, such as *Bmp4* and calmodulin, which are thought to be key determinants of jaw morphology in African cichlids and Galapagos finches are notably not differentially expressed in pupfishes with distinctive jaw phenotypes (Fig. 6). While it is very possible that these genes could be differentially expressed at time points not sampled, our data suggest that the sources of skull diversity in pupfishes differs from what has been shown for these other vertebrate taxa. We cannot rule out the possibility that RNA-seq is either not sensitive enough to pick up a very slight change in the expression of these genes, or that these genes are post-transcriptionally modified.

Among the genes that were identified by RNA-seq as possibly related to jaw diversification in pupfishes are several that code for molecules functioning in growth factor signaling and cytokine signaling. These functional groups are particularly interesting because they point to genes of interest as well as possible cellular-developmental processes underlying the origins of jaw morphological diversity. Below we outline three hypotheses that emerge from our RNA-seq data.

#### Wnt signaling

We found multiple *wnt* ligands to be differentially expressed among pupfish taxa at 48 hpf. We note that these genes would not have been identified had we applied a 1.5 fold change threshold, though GSEA analysis did identify enrichment of Wnt signaling in some comparisons. At 48 hpf, neural crest cells contributing to jaw development are relatively undifferentiated and aggregated in the pharyngeal pouches of the pupfish head. After this stage, embryos experience a period of rapid growth and formation of cranial cartilages.

Wnt signaling plays an established role in bone development, regulating the growth, differentiation, and functioning of bone remodeling cells such as osteoblasts [54, 76]. Modifications to Wnt signaling have been shown to affect bone mass and homeostasis in general [76], and to affect craniofacial development in particular [25–27, 29, 51, 77]. Chief among genes we identified as overexpressed in the scale-biter is *wnt11*, which is identified as the gene affected in zebrafish *silberblick* mutants [51]. Zebrafish lacking functional Wnt11 show dramatic reductions to the anterior skull elements, the same bony skull elements most different among species of pupfishes. Interestingly, the phenotypic effect of the Wnt11 gene in zebrafish is modified by the transmembrane protein Tmem88 [78]. Zebrafish double morpholino knockdowns targeting both *wnt11* and *tmem88* expression have more extreme phenotypes. In pupfishes, *tmem88* is underexpressed in the durophage at the same stage that *wnt11* is overexpressed in the scale-biter.

Wnt signaling has been shown to be associated with jaw diversification in African cichlids [26, 79], with the evolution of a fused maxilla in the bird beak [27], and with the specification and morphogenesis of jaw structures in mice [25, 77]. Thus Wnt signaling is emerging as an important source of craniofacial variation in wild taxa. With the exception of *wnt2b*, Wnt ligands were consistently overexpressed in the scale-biter and underexpressed in the durophage (Fig. 4). This raises the hypothesis of whether Wnt signaling is differentially regulated in the scale-biter and durophage taxa at early embryonic stages, a result further supported by GSEA results. That we find multiple Wnt ligands all differentially expressed at the same stage suggests that a process upstream of Wnt may be differentially regulated. Hedgehog signaling often regulates and is co-regulated by Wnt ligand production in other systems, and is thus an obvious candidate. In the head, Wnt signaling is known to interact with a frontal nasal cell proliferative zone (FEZ) marked by adjacent *shh*/*fgf8* expression domains [15, 27, 80]. Thus multiple avenues are available for future work experimentally manipulating Wnt ligands and exploring how Wnt interactions with *shh*/*fgf8* expression are modified (or not) among species of pupfishes.

#### Insulin-like growth factor signaling

Insulin-like growth factors (IGF-1 and IGF-2) are two of the most abundant growth factors in bone, inducing a number of transcriptional responses in myoblasts, chondrocytes, and osteoblasts that affect cellular differentiation, activity, and rates of bone deposition and homeostasis [54, 81]. IGF-1 has been associated with variation in dog breed size [82], but a function for IGF-1 signaling in skull formation is less well understood (though see [83, 84]). In the extracellular matrix, Igf proteins bind to Igf binding proteins, and the cellular responses to Igf proteins are strongly dependent on the presence of different Igf binding proteins [54]. We found two binding proteins, *igfbp2* and *igfbp5-like* (most probably one of two *igfbp5* paralogs), to be greatly overexpressed in the scale-biter at all stages. Transcript abundance of *igfbp2* in the scale-biter was more than 2-fold that of other species indicating substantial up-regulation and both genes would have been identified with a higher DE threshold, highlighting the extreme overexpression of these genes in the scale-biter. Further supporting a potential role for Igf signaling is that GSEA identified enrichment of Igf signaling in genes overexpressed in the scale-biter (Additional file 9: Table S6). The effects of Igf binding proteins on bone growth and homeostasis are typically complex and depend on numerous factors [54]. In mammalian systems *igfbp2* has been associated with both negative regulation of bone mass [85, 86] and positive stimulation of osteoblast differentiation and activity [87], while *igfbp5* has typically been associated with increased bone deposition [54]. Interestingly, we also find a number of genes related to metabolism and lipid transport that would be concordant with altered cellular responses in response to modified Igf signaling among species. Given the critical role Igf signaling plays in bone development and remodeling, and the dramatic overexpression of these two Igf binding proteins in the scale-biter, this is an obvious candidate for further study.

#### Cytokine and Chemokine signaling

Our observation that multiple cytokine/chemokine-related signaling molecules are differentially expressed among species of pupfishes is interesting because it provides a potential link between the cellular inflammation response and bone homeostasis such as occurs during pathologies like osteoporosis [88]. In our dataset, genes linked to inflammation included tumor necrosis factor family members, cytokines, interleukins and other immune cell stimulatory molecules, as well as inflammation activated intracellular molecules such as a number of Nod-like receptor paralogs (Tables 1, 2, 3 and 4; Additional file 1: Tables S2-S26).

In addition to roles in immune function and inflammation, cytokine signaling plays a critical role during non-pathological bone homeostasis [54]. Tumor necrosis factor and interleukin molecules mediate signaling among osteoblasts and osteoclasts, and play established roles affecting osteoclast differentiation and functioning [54, 88, 89]. We found many cytokine genes to be DE at 48 and 96 hpf, prior to bone formation, perhaps suggesting modifications to osteoclast differentiation. Our data further showed that a number of genes expressed by osteoclasts including calcitonin receptors (*calcr* and *calcrl*) and tartrate resistant acid phosphatase (*acp5*) tended to be overexpressed in the scale-biter further lending support to the idea that either osteoclast number or functioning may vary among species of pupfishes. The central role osteoclasts play in bone remodeling makes this an intriguing new avenue for research.

Studies of transcriptomic responses to bone loading following exercise have also implicated a dominant role for cytokine signaling in bone homeostasis [61, 74, 90]. Many of the genes identified as differentially expressed in our dataset are orthologs and gene family members of genes that have also been found to be differentially expressed in the pharyngeal jaws of adult cichlid fishes feeding on hard-shelled prey or soft food, and in the bones of adult mice undergoing bone remodeling following exercise [61, 74, 91]. Thus, differential expression of a number of cytokine signaling genes among pupfishes might suggest a connection between the cellular mechanisms of developmental plasticity and the cellular mechanisms underlying evolutionary divergence.

### Constitutive differential expression of genes

Careful consideration of developmental stage is commonly believed to be critical for identifying modifications to gene expression associated with phenotypic change. This can provide a dilemma for researchers deciding how fine to sample across development (a concern especially relevant to research on non-model genetic organisms since samples are often relatively labor intensive to obtain). An alternative model considers that once a gene is expressed in an organ, it may continue to be expressed in that organ for a long period of time (e.g. constitutive expression). We investigated this by identifying genes DE at more than one stage and found that while approximately 80% of the genes in the intersection sets were DE at only a single stage, a sizeable percentage of genes (~20%) tended towards being over- or under-expressed in either the scale-biter or durophage at multiple stages (Fig. 6).

Periods between our sampling stages were as few as two days and as much as a week. We reasoned, therefore, that when a gene was differentially expressed at more than one stage, this was good evidence that that gene was differentially expressed among species for a long block of time. We refer to these genes as constitutively differentially expressed genes since they followed a pattern of being over- or underexpressed among species through embryogenesis and juvenile growth. The presence of these constitutively DE genes would suggest that in some instances fine sampling of developmental time may be unnecessary to identify a non-negligible percentage of genes as DE. Furthermore, the true percentage of constitutively differentially expressed genes is almost certainly higher than what we report here since we highlight in this paper patterns from only those genes that appeared in two or more of our intersection sets, but we noted that many additional genes also showed this pattern of constitutive expression in our dataset. We suspect that this pattern of constitutive differential expression may be a dominant pattern for many of the genes identified by us to be differentially expressed among closely related pupfish taxa.

We want to stress that while many genes appear to be constitutively differentially expressed, a much greater percentage (~80%) of genes were DE at only 1 or 2 stages. Gene expression is known to be dynamic during embryonic development when body plans and organs are being patterned. Thus different classes of genes may be more or less likely to be constitutively differentially expressed, and some genes, DE at only a single stage, may be evolutionarily important.

### Other traits

The pupfishes in this study differ not only in jaw morphology, but also traits such as behavior and coloration [12, 13, 92, 93]. Undoubtedly some of the transcriptional differences that we identified are related to these and other traits. For example, GSEA identified enrichment of neuronal pathways at embryonic stages (and presumptive brain tissue was necessarily included in these early embryonic samples) highlighting to us that not every expression difference we identify is related to jaw morphology. Determining which genes are related to jaw morphology, however, is complicated by interactions among different tissues. For instance, signaling centers located in the developing brain are known to play a role in craniofacial development [16].

As a further example, the inland species differ dramatically in coloration in both wild and laboratory conditions. Adult male scale-biters are uniformly black, while durophage males and females have a milky white body coloration. Vertebrate jaws and pigment cells both develop from migratory neural crest cells, and two genes differentially expressed in our data, endothelin receptor b (*ednrb*) and macrophage colony stimulating factor 1 (*csf1b*), are implicated to affect both jaw/bone development and melanophore number in zebrafish [59, 94]. All three pupfishes have melanophore cells (black) and leucophore cells (white), but whether these coloration differences result from changes to cell number, distribution, or by some other mechanism is unknown. These same genes may or may not play a role in color differences among species of pupfishes (or jaws for that matter); we mention these only to emphasize that some transcriptional variation in our dataset may affect traits other than jaws or have pleiotropic effects on multiple traits that differ among pupfishes.

## Conclusions

We used RNA-seq to identify differentially expressed genes that may affect jaw development in pupfishes. Our data indicated that a number of molecules related to cell proliferation and differentiation, growth, bone development, and craniofacial form were differentially expressed prior to the appearance of morphological differences among species. In particular we identified a number of growth factor genes that are strong candidates for future research into the origins of jaw diversity in this group.

Our findings are concordant with recent work in birds, fishes, and mammals that have shown diverse mechanisms contributing to skull variation in different clades [11, 19, 20, 26–28, 95]. For complex traits such as skull morphology, what we really want to know is how the evolutionary tinkering of conserved developmental processes can produce macroevolutionary patterns of phenotypic diversity. Our data have identified a number of genes that play roles in modifying conserved developmental processes during skull development. Future work linking modifications of gene expression as shown here with changes in cellular-developmental processes such as cell proliferation, apoptosis, and differentiation will provide further insight into how a complex trait such as the vertebrate skull is modified during the early stages of speciation and ecological differentiation.

## Additional files

Additional file 1: Table S1. Includes STAR read mapping statistics. (DOCX 131 kb)

Additional file 2: Figure S1. Phylogenetic relationships among San Salvador Island *Cyprinodon* taxa. Shown are maximum likelihood phylogenies built using RAxML under either a k-means partitioning scheme implemented by Partition Finder or a single partition scheme [49], and by applying either a GTRGAMMA or GTRCAT model. Note the general overall congruence across trees built using different assumptions, and that in each case the ML tree identifies the marine omnivore as and outgroup to a monophyletic San Salvador clade. (PDF 115 kb)

Additional file 3: Figure S2. Heatmap of sample by sample correlations (Pearson’s r) shows both (1) dramatic differences in gene expression among stages and (2) that samples are highly correlated within each stage. Dendrogram represents hierarchical clustering tree of samples at all four stages based on log2 transformed RPKM gene expression values. Note the four major clusters corresponding to stage that appear as blocks of high correlation (red) in the heatmap. The dendrogram tips are colored by taxa showing that within each stage samples cluster by taxa. Durophage = red, Inland omnivore = blue, Marine omnivore = green, Scale-biter = purple. (PDF 2400 kb)

Additional file 4: Figure S3. Principal component plots show separation of taxa by gene expression along the first 3–4 PC axes. Shown are the first 3 (48 hpf) or 4 (96 hpf, 8 dpf, 15 dpf) PC axes for each stage. Note how different PC axes separate taxa. For instance at 96 hpf PC2 largely distinguishes the scale-biter samples from the other taxa, while PC4 largely distinguishes the durophage and inland omnivore samples. (PDF 175 kb)

Additional file 5: Table S2. GSEA results for Hallmark gene sets along PC axes at each stage. Excel table provides results from GSEA enrichment analysis for Hallmark gene sets along PC axes at each stage. Genes were pre-ranked by loadings on each axis prior to analysis. Human gene identifiers were used, and genes without an identifiable human ortholog were excluded from analysis. (XLS 98 kb)

Additional file 6: Table S3. GSEA results for Canonical Pathways gene sets along PC axes at each stage. Excel table provides results from GSEA enrichment analysis for Canonical Pathways gene sets along PC axes at each stage. Genes were pre-ranked by loadings on each axis prior to analysis. Human gene identifiers were used, and genes without an identifiable human ortholog were excluded from analysis. (XLS 552 kb)

Additional file 7: Table S4. GSEA results for Hallmark gene sets for genes over- or underexpressed in the scale-biter. Excel table provides results from GSEA enrichment analysis for Hallmark gene sets in genes differentially expressed in the scale-biter. Genes were pre-ranked by logFC in the scale-biter relative to all other taxa. Human gene identifiers were used, and genes without an identifiable human ortholog were excluded from analysis. (XLS 49 kb)

Additional file 8: Table S5. GSEA results for Hallmark gene sets for genes over- or underexpressed in the durophage. Excel table provides results from GSEA enrichment analysis for Hallmark gene sets in genes differentially expressed in the durophage. Genes were pre-ranked by logFC in the scale-biter relative to all other taxa. Human gene identifiers were used, and genes without an identifiable human ortholog were excluded from analysis. (XLS 49 kb)

Additional file 9: Table S6. GSEA results for Canonical Pathways gene sets for genes over- or underexpressed in the scale-biter. Excel table provides results from GSEA enrichment analysis for Canonical Pathways gene sets in genes differentially expressed in the scale-biter. Genes were pre-ranked by logFC in the scale-biter relative to all other taxa. Human gene identifiers were used, and genes without an identifiable human ortholog were excluded from analysis. (XLS 162 kb)

Additional file 10: Table S7. GSEA results for Canonical Pathways gene sets for genes over- or underexpressed in the durophage. Excel table provides results from GSEA enrichment analysis for Canonical Pathways gene sets in genes differentially expressed in the durophage. Genes were pre-ranked by logFC in the scale-biter relative to all other taxa. Human gene identifiers were used, and genes without an identifiable human ortholog were excluded from analysis. (XLS 105 kb)

Additional file 11: Figure S4. Identification of Intersection Sets. Venn diagrams showing the selection of intersection sets of genes differentially expressed in either the scale-biter (*C. desquamator*) or durophage (*C. brontotheroides*) at each stage. Numbers correspond to the number of genes in each set. Genes in the middle region are differentially expressed in all comparisons and are considered the intersection set of genes most likely to contribute to the derived skull morphology of the scale-biter and durophage respectively. (PDF 446 kb)

Additional file 12: Figure S5. Histograms of log2 fold change values for genes differentially expressed (FDR ≤ 0.1) at 48 hpf. Histograms of log2 fold change values for genes differentially expressed (FDR ≤ 0.1) at 48 hpf in all pairwise comparisons. Most genes are differentially expressed by 1.2–1.5 fold difference, with a much smaller number of genes DE by greater than 1.5 fold indicating a modest change to the magnitude at which most genes are DE. Insets highlight genes differentially expressed at log2 fold change less than 2. (PDF 271 kb)

Additional file 13: Figure S6. Histograms of log2 fold change values for genes differentially expressed (FDR ≤ 0.1) at 96 hpf. Histograms of log2 fold change values for genes differentially expressed (FDR ≤ 0.1) at 96 hpf in all pairwise comparisons. Most genes are differentially expressed by 1.2–1.5 fold difference, with a much smaller number of genes DE by greater than 1.5 fold indicating a modest change to the magnitude at which most genes are DE. Insets highlight genes differentially expressed at log2 fold change less than 2. (PDF 273 kb)

Additional file 14: Figure S7. Histograms of log2 fold change values for genes differentially expressed (FDR ≤ 0.1) at 8 dpf. Histograms of log2 fold change values for genes differentially expressed (FDR ≤ 0.1) at 8 dpf in all pairwise comparisons. Most genes are differentially expressed by 1.2–1.5 fold difference, with a much smaller number of genes DE by greater than 1.5 fold indicating a modest change to the magnitude at which most genes are DE. But compare to embryonic stages 48 hpf and 96 hpf there are many more genes DE by greater than 1.5 fold. Insets highlight genes differentially expressed at log2 fold change less than 2. (PDF 271 kb)

Additional file 15: Figure S8. Histograms of log2 fold change values for genes differentially expressed (FDR ≤ 0.1) at 15 dpf. Histograms of log2 fold change values for genes differentially expressed (FDR ≤ 0.1) at 15 dpf in all pairwise comparisons. Most genes are differentially expressed by 1.2–1.5 fold difference, with a much smaller number of genes DE by greater than 1.5 fold indicating a modest change to the magnitude at which most genes are DE. But compare to embryonic stages 48 hpf and 96 hpf there are many more genes DE by greater than 1.5 fold. Insets highlight genes differentially expressed at log2 fold change less than 2. (PDF 265 kb)

Additional file 16: Figure S9. Heatmap of genes in both the scale-biter and durophage intersection sets. Differentially expressed genes are over- or underexpressed in a single taxon. Shown are heatmaps of all genes in both the scale-biter and durophage intersection sets at 48 hpf (**A**) and at 96 hpf, 8 dpf, and 15 dpf (**B**). Note how the data fall into four main clusters at each stage that are easily visualized by eye corresponding to genes over or underexpressed in either the scale-biter or durophage respectively. In contrast, genes are not typically differentially expressed in both the scale-biter and durophage taxa. For instance, the plot in **A** shows genes overexpressed in the scale-biter, and note that these same genes are similarly expressed among the other three taxa. (PDF 12001 kb)

Additional file 17: Table S8. Genes in scale-biter intersection set at 48 hpf. This is a comma separated table of the genes in the 48 hpf scale-biter intersection set. Given are edgeR results for each pairwise comparison. Columns indicating whether a gene is included in the intersection set at a threshold of 1.5 or 2 fold are provided. (CSV 143 kb)

Additional file 18: Table S9. Genes in scale-biter intersection set at 96 hpf. This is a comma separated table of the genes in the 96 hpf scale-biter intersection set. Given are edgeR results for each pairwise comparison. Columns indicating whether a gene is included in the intersection set at a threshold of 1.5 or 2 fold are provided. (CSV 65 kb)

Additional file 19: Table S10. Genes in scale-biter intersection set at 8 dpf. This is a comma separated table of the genes in the 8 dpf scale-biter intersection set. Given are edgeR results for each pairwise comparison. Columns indicating whether a gene is included in the intersection set at a threshold of 1.5 or 2 fold are provided. (CSV 48 kb)

Additional file 20: Table S11. Genes in scale-biter intersection set at 15 dpf. This is a comma separated table of the genes in the 15 dpf scale-biter intersection set. Given are edgeR results for each pairwise comparison. Columns indicating whether a gene is included in the intersection set at a threshold of 1.5 or 2 fold are provided. (CSV 91 kb)

Additional file 21: Table S12 Genes in durophage intersection set at 48 hpf. This is a comma separated table of the genes in the 48 hpf durophage intersection set. Given are edgeR results for each pairwise comparison. Columns indicating whether a gene is included in the intersection set at a threshold of 1.5 or 2 fold are provided. (CSV 76 kb)

Additional file 22: Table S13. Genes in durophage intersection set at 96 hpf. This is a comma separated table of the genes in the 96 hpf durophage intersection set. Given are edgeR results for each pairwise comparison. Columns indicating whether a gene is included in the intersection set at a threshold of 1.5 or 2 fold are provided. (CSV 26 kb)

Additional file 23: Table S14. Genes in durophage intersection set at 8 dpf. This is a comma separated table of the genes in the 8 dpf durophage intersection set. Given are edgeR results for each pairwise comparison. Columns indicating whether a gene is included in the intersection set at a threshold of 1.5 or 2 fold are provided. (CSV 22 kb)

Additional file 24: Table S15. Genes in durophage intersection set at 15 dpf. This is a comma separated table of the genes in the 15 dpf durophage intersection set. Given are edgeR results for each pairwise comparison. Columns indicating whether a gene is included in the intersection set at a threshold of 1.5 or 2 fold are provided. (CSV 13 kb)

Additional file 25: Table S16. GOstats results for scale-biter intersection set. This is an excel table of providing GOstats overrepresentation enrichment analysis results for each of the 4 scale-biter intersection sets. (XLS 87 kb)

Additional file 26: Table S17. GOstats results for durophage intersection set. This is an excel table of providing GOstats overrepresentation enrichment analysis results for each of the 4 durophage intersection sets. (XLS 53 kb)

Additional file 27: Table S18. WebGestalt results for scale-biter intersection set. This is an excel table of providing WebGestalt overrepresentation enrichment analysis results for each of the 4 scale-biter intersection sets. Human symbols were used for analysis and genes without orthology assignment were excluded. (XLS 74 kb)

Additional file 28: Table S19. WebGestalt results for durophage intersection set. This is an excel table of providing WebGestalt overrepresentation enrichment analysis results for each of the 4 durophage intersection sets. Human symbols were used for analysis and genes without orthology assignment were excluded. (XLS 66 kb)

Additional file 29: Table S20. DAVID results for scale-biter intersection set. This is an excel table of providing DAVID overrepresentation enrichment analysis results for each of the 4 scale-biter intersection sets. ZFIN ids were used for analysis and genes without orthology assignment were excluded. (XLS 164 kb)

Additional file 30: Table S21. DAVID results for durophage intersection set. This is an excel table of providing DAVID overrepresentation enrichment analysis results for each of the 4 durophage intersection sets. ZFIN ids were used for analysis and genes without orthology assignment were excluded. (XLS 90 kb)

Additional file 31: Table S22. edgeR results table for identification of DE genes. This is a tab delimited table of the results from pair-wise comparisons among taxa at each stage to test for differential expression of genes using edgeR. GLM models were built for each stage and TMM normalization was calculated and applied at each stage. Genes filtered in a given stage are represented by NA. (GZ 10772 kb)

## Abbreviations

cpm: Read counts per million
DE: Differentially expressed
dpf: Days post fertilization
GLM: Generalized linear model
GSEA: Gene set enrichment analysis (as implemented in Broad Institutes GSEA program)
hpf: Hours post fertilization
LogFC: Logarithimic (base 2) fold-change
MGI: Mouse Genome Informatics Database
ML: Maximum likelihood
PC: Principal component
PheGenI: NCBI Phenotype-Genotype Integrator
ppt: Parts per thousand (salinity)
ZFIN: Zebrafish Model Organism Database
