# Supplementary material for: The Cyprinodon variegatus genome reveals gene expression changes underlying differences in skull morphology among closely related species
Source: BMC Genomics. 2017 May 30;18:424. doi: 10.1186/s12864-017-3810-7 (PMC5450241; doi:10.1186/s12864-017-3810-7)
Supplement: Supplementary file 1 — Includes STAR read mapping statistics. (DOCX 131 kb) [file 12864_2017_3810_MOESM1_ESM.docx]

| Table S1. Illumina sequencing and STAR mapping to genome statistics | | | | | | | | | | | |
| --- | --- | --- | --- | --- | --- | --- | --- | --- | --- | --- | --- |
|  |  | Library Size | Mean read length | Mean Mapped length | Uniq. Mapped Reads^1^ | | Mismatch rate | Deletion rate | Insertion Rate | Num. Reads mapped to multiple loci | |
| **48 hpf** | |  |  |  |  |  |  |  |  |  |  |
|  | Durophage Replicate 1 | 30623011 | 97 | 96.48 | 28551205 | (96.48) | 0.93 | 0.05 | 0.03 | 936657 | (3.06) |
|  | Durophage Replicate 2 | 26343284 | 138 | 137 | 24549772 | (93.19) | 0.92 | 0.07 | 0.04 | 688702 | (2.61) |
|  | Durophage Replicate 3 | 41567278 | 125 | 124.44 | 38685529 | (93.07) | 0.88 | 0.06 | 0.03 | 1133820 | (2.73) |
|  | Durophage Replicate 4 | 24337107 | 138 | 137.44 | 22695691 | (93.26) | 0.91 | 0.07 | 0.04 | 634744 | (2.61) |
|  | Inland Omnivore Replicate 1 | 39810934 | 97 | 96.98 | 37012044 | (92.97) | 0.95 | 0.05 | 0.03 | 1261913 | (3.17) |
|  | Inland Omnivore Replicate 2 | 48591822 | 133 | 132.04 | 45504090 | (93.65) | 0.84 | 0.06 | 0.04 | 1268545 | (2.61) |
|  | Inland Omnivore Replicate 3 | 39019101 | 114 | 114 | 36453719 | (93.43) | 1.02 | 0.06 | 0.03 | 1114942 | (2.86) |
|  | Inland Omnivore Replicate 4 | 42813136 | 138 | 137.5 | 40056586 | (93.56) | 0.89 | 0.06 | 0.04 | 1092799 | (2.55) |
|  | Marine Omnivore Replicate 1 | 43226250 | 116 | 115.77 | 40329252 | (93.3) | 1 | 0.06 | 0.04 | 1262367 | (2.92) |
|  | Marine Omnivore Replicate 2 | 35557860 | 111 | 110.83 | 33153522 | (93.24) | 0.9 | 0.06 | 0.04 | 1060624 | (2.98) |
|  | Marine Omnivore Replicate 3 | 42035564 | 108 | 107.64 | 39159934 | (93.16) | 0.98 | 0.06 | 0.03 | 1307777 | (3.11) |
|  | Marine Omnivore Replicate 4 | 44330878 | 125 | 124.94 | 41487578 | (93.59) | 0.89 | 0.06 | 0.04 | 1209906 | (2.73) |
|  | Scale-biter Replicate 1 | 54410403 | 116 | 115.38 | 50898745 | (93.55) | 1 | 0.06 | 0.03 | 1548615 | (2.85) |
|  | Scale-biter Replicate 2 | 42104342 | 111 | 110.37 | 39336801 | (93.43) | 0.89 | 0.06 | 0.03 | 1234385 | (2.93) |
|  | Scale-biter Replicate 3 | 38026040 | 131 | 130.05 | 35401223 | (93.1) | 0.89 | 0.08 | 0.05 | 1056825 | (2.78) |
|  | Scale-biter Replicate 4 | 43277454 | 97 | 96.98 | 40480895 | (93.54) | 0.91 | 0.05 | 0.03 | 1316609 | (3.04) |
| **96 hpf** | |  |  |  |  |  |  |  |  |  |  |
|  | Durophage Replicate 1 | 32220564 | 96 | 96.24 | 30044926 | (93.25) | 0.95 | 0.05 | 0.03 | 992672 | (3.08) |
|  | Durophage Replicate 2 | 44262078 | 107 | 106.91 | 41116237 | (92.89) | 0.99 | 0.07 | 0.04 | 1382354 | (3.12) |
|  | Durophage Replicate 3 | 28062508 | 131 | 130.03 | 26217343 | (93.42) | 0.91 | 0.07 | 0.04 | 757530 | (2.7) |
|  | Durophage Replicate 4 | 44063213 | 115 | 114.84 | 41069411 | (93.21) | 1.01 | 0.07 | 0.04 | 1289087 | (2.93) |
|  | Inland Omnivore Replicate 1 | 35895791 | 97 | 97.36 | 33452990 | (93.19) | 0.94 | 0.06 | 0.04 | 1121840 | (3.13) |
|  | Inland Omnivore Replicate 2 | 48733723 | 123 | 122.88 | 45594950 | (93.56) | 0.9 | 0.07 | 0.04 | 1343135 | (2.76) |
|  | Inland Omnivore Replicate 3 | 31535106 | 138 | 137.36 | 29529976 | (93.64) | 0.9 | 0.07 | 0.04 | 825050 | (2.62) |
|  | Inland Omnivore Replicate 4 | 31367484 | 131 | 130.11 | 29306955 | (93.43) | 0.89 | 0.08 | 0.04 | 854449 | (2.72) |
|  | Marine Omnivore Replicate 1 | 46027847 | 111 | 110.5 | 42893780 | (93.19) | 0.91 | 0.07 | 0.04 | 1424351 | (3.09) |
|  | Marine Omnivore Replicate 2 | 40958156 | 130 | 129.82 | 38322127 | (93.56) | 0.88 | 0.07 | 0.04 | 1106436 | (2.7) |
|  | Marine Omnivore Replicate 3 | 41997633 | 97 | 97.04 | 39255845 | (93.47) | 0.92 | 0.06 | 0.03 | 1289780 | (3.07) |
|  | Marine Omnivore Replicate 4 | 40269810 | 116 | 115.5 | 37532004 | (93.2) | 1.02 | 0.07 | 0.04 | 1204128 | (2.99) |
|  | Scale-biter Replicate 1 | 44731712 | 124 | 123.89 | 41846030 | (93.55) | 0.89 | 0.07 | 0.04 | 1218889 | (2.72) |
|  | Scale-biter Replicate 2 | 31180817 | 105 | 105.2 | 29081701 | (93.27) | 0.99 | 0.06 | 0.04 | 958749 | (3.07) |
|  | Scale-biter Replicate 3 | 44673757 | 98 | 98 | 41634168 | (93.2) | 0.93 | 0.07 | 0.04 | 1393852 | (3.12) |
|  | Scale-biter Replicate 4 | 42538488 | 132 | 131.41 | 39823888 | (93.62) | 0.86 | 0.08 | 0.05 | 1145755 | (2.69) |
| **8 dpf** | |  |  |  |  |  |  |  |  |  |  |
|  | Durophage Replicate 1 | 51123118 | 133 | 132.12 | 47677926 | (93.26) | 0.83 | 0.05 | 0.04 | 1519069 | (2.97) |
|  | Durophage Replicate 2 | 44632685 | 125 | 124.35 | 41548257 | (93.09) | 0.87 | 0.06 | 0.04 | 1345195 | (3.01) |
|  | Durophage Replicate 3 | 34669935 | 113 | 112.92 | 32255420 | (93.04) | 1 | 0.05 | 0.03 | 1107081 | (3.19) |
|  | Durophage Replicate 4 | 35014190 | 110 | 109.4 | 32534843 | (92.92) | 0.9 | 0.06 | 0.03 | 1130833 | (3.23) |
|  | Inland Omnivore Replicate 1 | 45030595 | 116 | 115.33 | 41961637 | (93.18) | 0.99 | 0.06 | 0.03 | 1395033 | (3.1) |
|  | Inland Omnivore Replicate 2 | 50418643 | 132 | 131.81 | 47087514 | (93.39) | 0.83 | 0.06 | 0.03 | 1462195 | (2.9) |
|  | Inland Omnivore Replicate 3 | 46164423 | 125 | 124 | 42987120 | (93.12) | 0.88 | 0.06 | 0.04 | 1437520 | (3.11) |
|  | Inland Omnivore Replicate 4 | 34509189 | 131 | 130.16 | 32190167 | (93.28) | 0.87 | 0.06 | 0.04 | 1035333 | (3.00) |
|  | Marine Omnivore Replicate 1 | 38890897 | 138 | 137 | 36237121 | (93.18) | 0.88 | 0.06 | 0.04 | 1174386 | (3.02) |
|  | Marine Omnivore Replicate 2 | 44011598 | 131 | 130.5 | 41036411 | (93.24) | 0.85 | 0.06 | 0.03 | 1331701 | (3.03) |
|  | Marine Omnivore Replicate 3 | 33885365 | 98 | 97.73 | 31506340 | (92.98) | 0.91 | 0.05 | 0.03 | 1223152 | (3.61) |
|  | Marine Omnivore Replicate 4 | 52503103 | 139 | 138.23 | 48920620 | (93.18) | 0.88 | 0.06 | 0.04 | 1516949 | (2.89) |
|  | Scale-biter Replicate 1 | 43350595 | 139 | 138.24 | 40209168 | (92.75) | 0.87 | 0.07 | 0.04 | 1354147 | (3.12) |
|  | Scale-biter Replicate 2 | 53031500 | 134 | 132.88 | 49242444 | (92.86) | 0.85 | 0.07 | 0.05 | 1660253 | (3.13) |
|  | Scale-biter Replicate 3 | 52635872 | 138 | 137.73 | 49143827 | (93.37) | 0.85 | 0.06 | 0.04 | 1519565 | (2.89) |
|  | Scale-biter Replicate 4 | 41208450 | 111 | 110.43 | 38297430 | (92.94) | 0.88 | 0.06 | 0.03 | 1399968 | (3.40) |
| **15 dpf** | |  |  |  |  |  |  |  |  |  |  |
|  | Durophage Replicate 1 | 37084747 | 111 | 110.39 | 34202803 | (92.23) | 0.93 | 0.07 | 0.04 | 1317398 | (3.55) |
|  | Durophage Replicate 2 | 46166499 | 108 | 107.74 | 42332988 | (91.7) | 1 | 0.06 | 0.04 | 1844816 | (4.00) |
|  | Durophage Replicate 3 | 32377904 | 132 | 131.29 | 29927809 | (92.43) | 0.9 | 0.08 | 0.05 | 1098203 | (3.39) |
|  | Durophage Replicate 4 | 48950909 | 134 | 133.42 | 45320338 | (92.58) | 0.86 | 0.07 | 0.05 | 1587475 | (3.24) |
|  | Inland Omnivore Replicate 1 | 40763659 | 111 | 110.98 | 37680189 | (92.44) | 0.93 | 0.07 | 0.04 | 1407203 | (3.45) |
|  | Inland Omnivore Replicate 2 | 33971482 | 110 | 109.6 | 31374005 | (92.35) | 0.91 | 0.06 | 0.04 | 1254366 | (3.39) |
|  | Inland Omnivore Replicate 3 | 43834577 | 108 | 107.88 | 40495679 | (92.38) | 0.96 | 0.06 | 0.04 | 1642490 | (3.75) |
|  | Inland Omnivore Replicate 4 | 48355935 | 109 | 108.53 | 44551341 | (92.13) | 0.99 | 0.07 | 0.04 | 1743411 | (3.61) |
|  | Marine Omnivore Replicate 1 | 41311050 | 125 | 124.74 | 38177142 | (92.41) | 0.91 | 0.06 | 0.04 | 1331645 | (3.22) |
|  | Marine Omnivore Replicate 2 | 47511808 | 108 | 107.68 | 43897034 | (92.39) | 0.99 | 0.06 | 0.04 | 1730771 | (3.64) |
|  | Marine Omnivore Replicate 3 | 43103146 | 134 | 133.71 | 39910440 | (92.59) | 0.87 | 0.07 | 0.05 | 1360280 | (3.16) |
|  | Marine Omnivore Replicate 4 | 49966400 | 134 | 133.53 | 46423945 | (92.91) | 0.86 | 0.07 | 0.04 | 1535893 | (3.07) |
|  | Scale-biter Replicate 1 | 45778579 | 134 | 133.86 | 42234006 | (92.26) | 0.89 | 0.08 | 0.05 | 1532109 | (3.35) |
|  | Scale-biter Replicate 2 | 45019262 | 126 | 125.43 | 41426563 | (92.02) | 0.92 | 0.08 | 0.05 | 1581401 | (3.51) |
|  | Scale-biter Replicate 3 | 51064646 | 117 | 116.66 | 47108632 | (92.25) | 1.01 | 0.07 | 0.04 | 1855552 | (3.63) |
|  | Scale-biter Replicate 4 | 40101073 | 108 | 107.4 | 36891255 | (92) | 1 | 0.06 | 0.03 | 1530757 | (3.82) |
| ^1^Percentage of reads in parentheses | | | | | | | | | | | |
