# Supplementary figures and images for: The Cyprinodon variegatus genome reveals gene expression changes underlying differences in skull morphology among closely related species
Source: BMC Genomics. 2017 May 30;18:424. doi: 10.1186/s12864-017-3810-7 (PMC5450241; doi:10.1186/s12864-017-3810-7)

# Partitioned Models

## GTR GAMMA

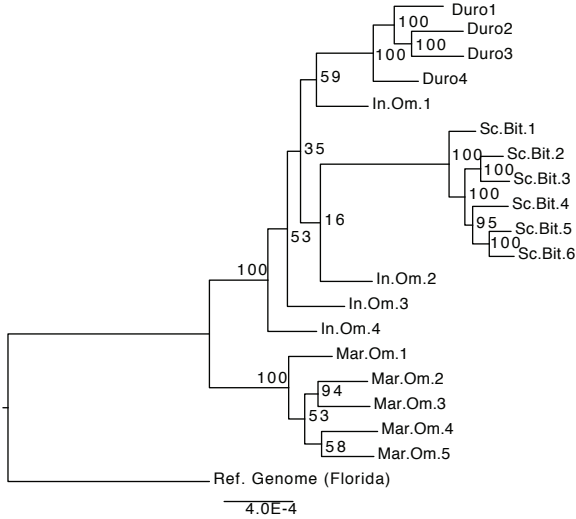

## GTR CAT

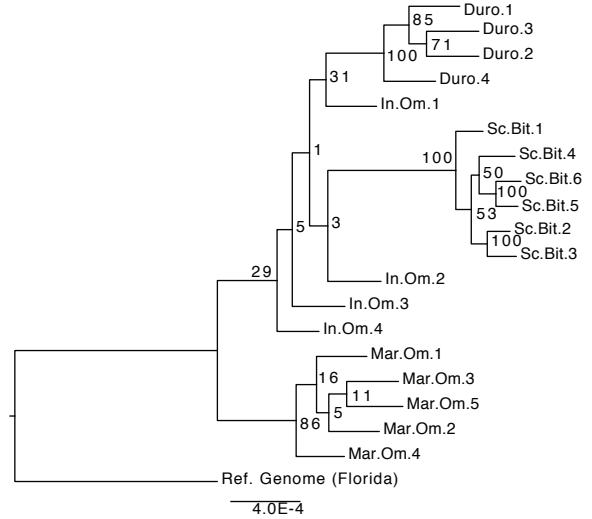

# Single Partition

## GTR GAMMA

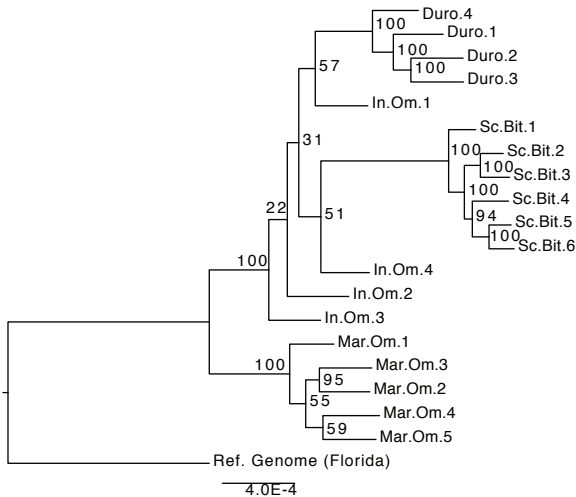

## GTR CAT

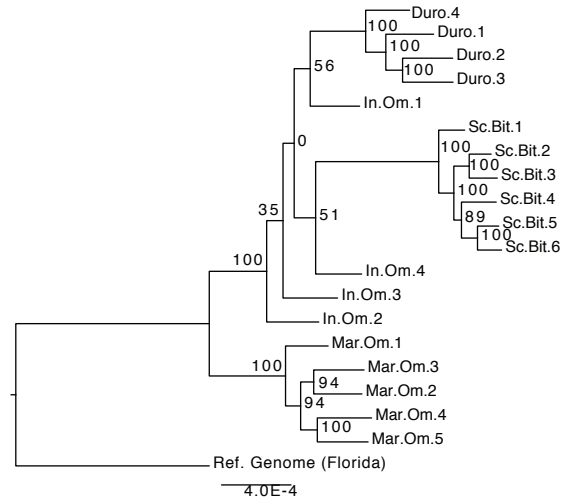

Figure S1

Supplement: Supplementary file 2 — Phylogenetic relationships among San Salvador Island Cyprinodon taxa. Shown are maximum likelihood phylogenies built using RAxML under either a k-means partitioning scheme implemented by Partition Finder or a single partition scheme [49], and by applying either a GTRGAMMA or GTRCAT model. Note the general overall congruence across trees built using different assumptions, and that in each case the ML tree identifies the marine omnivore as and outgroup to a monophyletic San Salvador clade. (PDF 115 kb) [file 12864_2017_3810_MOESM2_ESM.pdf]

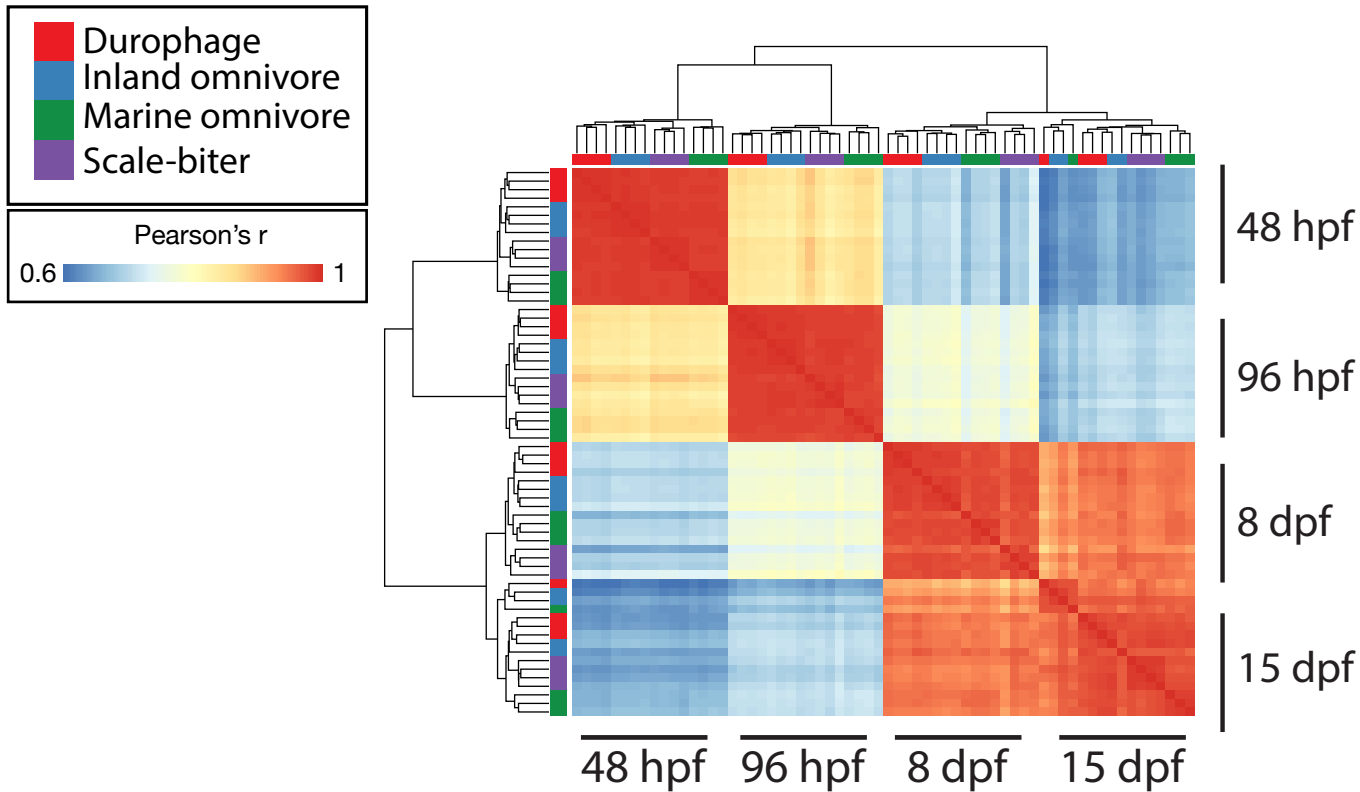

**Figure S2**

Supplement: Supplementary file 3 — Heatmap of sample by sample correlations (Pearson’s r) shows both (1) dramatic differences in gene expression among stages and (2) that samples are highly correlated within each stage. Dendrogram represents hierarchical clustering tree of samples at all four stages based on log2 transformed RPKM gene expression values. Note the four major clusters corresponding to stage that appear as blocks of high correlation (red) in the heatmap. The dendrogram tips are colored by taxa showing that within each stage samples cluster by taxa. Durophage = red, Inland omnivore = blue, Marine omnivore = green, Scale-biter = purple. (PDF 2400 kb) [file 12864_2017_3810_MOESM3_ESM.pdf]

48 hpf

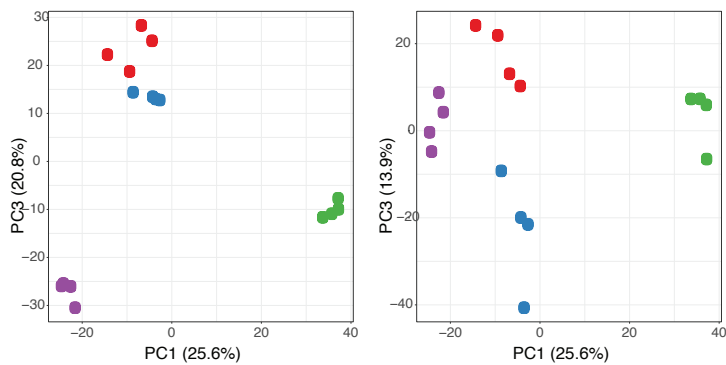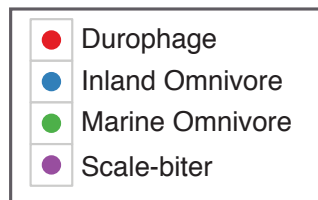

96 hpf

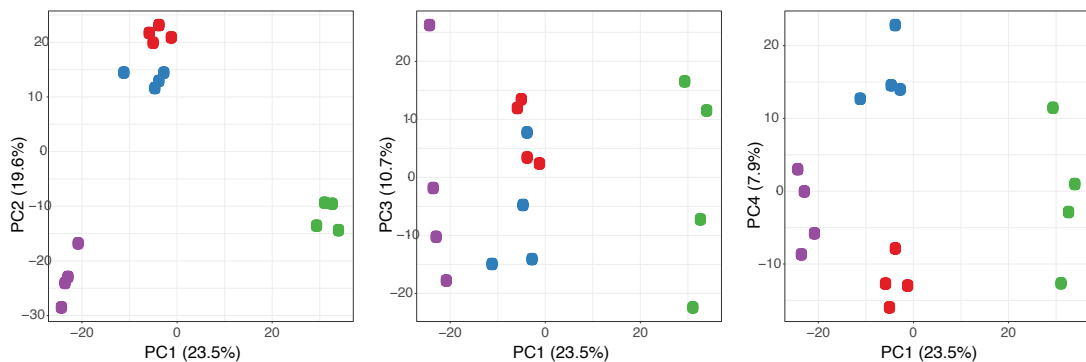

8 dpf

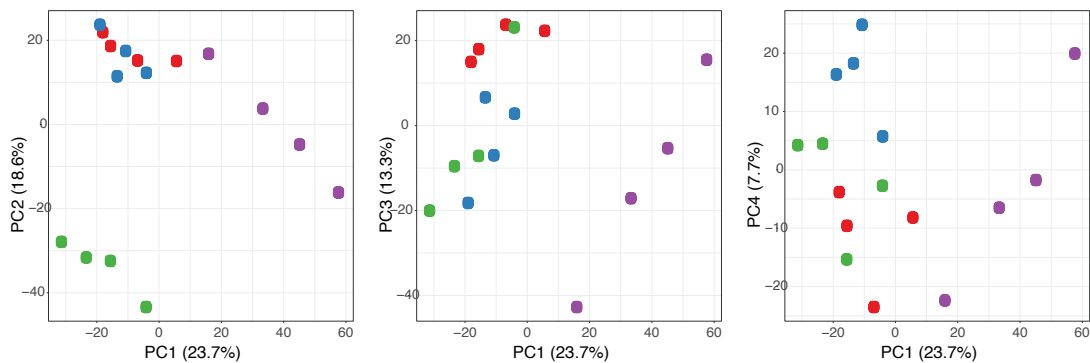

15 dpf

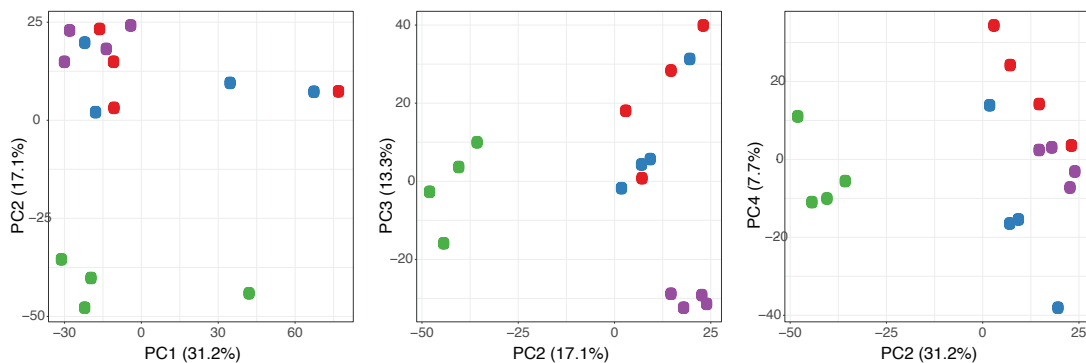

Figure S3

Supplement: Supplementary file 4 — Principal component plots show separation of taxa by gene expression along the first 3–4 PC axes. Shown are the first 3 (48 hpf) or 4 (96 hpf, 8 dpf, 15 dpf) PC axes for each stage. Note how different PC axes separate taxa. For instance at 96 hpf PC2 largely distinguishes the scale-biter samples from the other taxa, while PC4 largely distinguishes the durophage and inland omnivore samples. (PDF 175 kb) [file 12864_2017_3810_MOESM4_ESM.pdf]

## Scale-biter Intersections

## Durophage Intersections

**48 hpf**

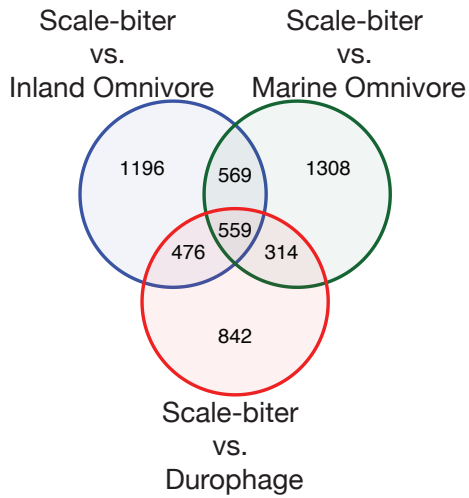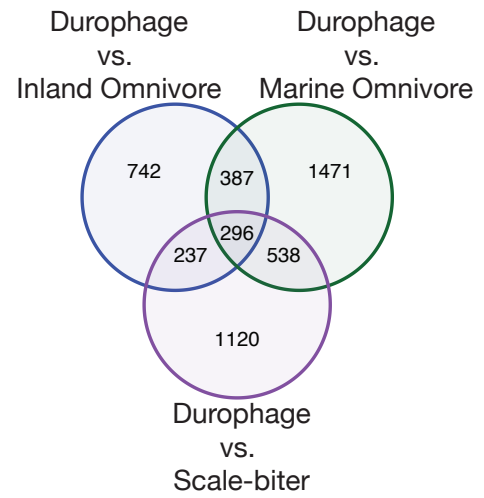

**96 hpf**

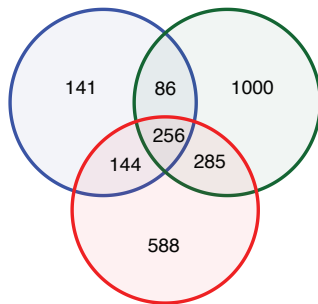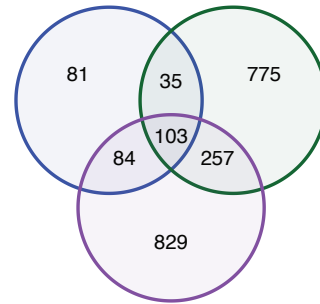

**8 dpf**

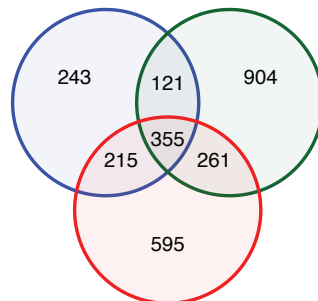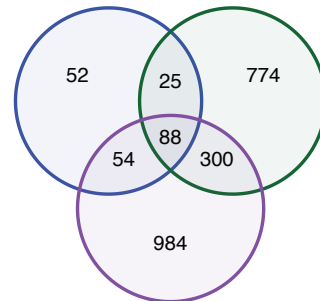

**15 dpf**

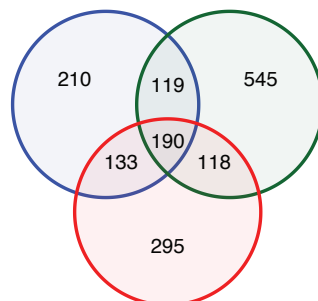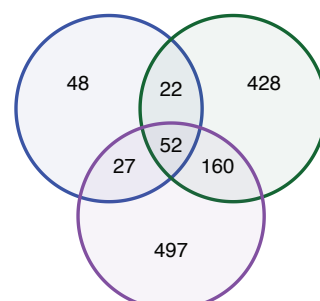

**Figure S4**

Supplement: Supplementary file 11 — Identification of Intersection Sets. Venn diagrams showing the selection of intersection sets of genes differentially expressed in either the scale-biter (C. desquamator) or durophage (C. brontotheroides) at each stage. Numbers correspond to the number of genes in each set. Genes in the middle region are differentially expressed in all comparisons and are considered the intersection set of genes most likely to contribute to the derived skull morphology of the scale-biter and durophage respectively. (PDF 446 kb) [file 12864_2017_3810_MOESM11_ESM.pdf]
