# Supplementary material for: The Cyprinodon variegatus genome reveals gene expression changes underlying differences in skull morphology among closely related species
Source: BMC Genomics. 2017 May 30;18:424. doi: 10.1186/s12864-017-3810-7 (PMC5450241; doi:10.1186/s12864-017-3810-7)
Supplement: Supplementary file 12 — Histograms of log2 fold change values for genes differentially expressed (FDR ≤ 0.1) at 48 hpf. Histograms of log2 fold change values for genes differentially expressed (FDR ≤ 0.1) at 48 hpf in all pairwise comparisons. Most genes are differentially expressed by 1.2–1.5 fold difference, with a much smaller number of genes DE by greater than 1.5 fold indicating a modest change to the magnitude at which most genes are DE. Insets highlight genes differentially expressed at log2 fold change less than 2. (PDF 271 kb) [file 12864_2017_3810_MOESM12_ESM.pdf]

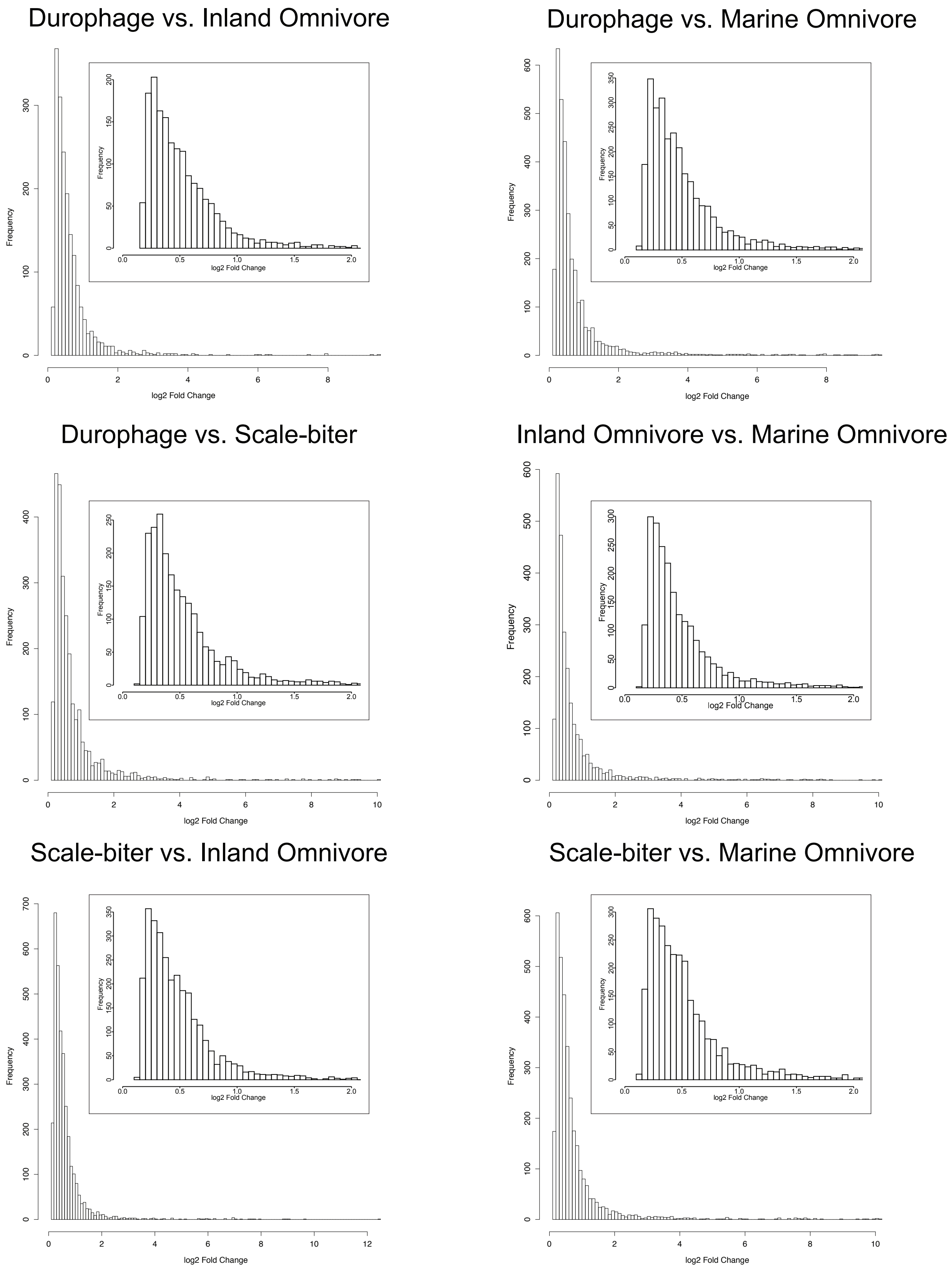

Durophage vs. Inland Omnivore
Durophage vs. Marine Omnivore
Durophage vs. Scale-biter
Inland Omnivore vs. Marine Omnivore
Scale-biter vs. Inland Omnivore
Scale-biter vs. Marine Omnivore
Frequency
log2 Fold Change

Figure S5
