# Supplementary material for: The Cyprinodon variegatus genome reveals gene expression changes underlying differences in skull morphology among closely related species
Source: BMC Genomics. 2017 May 30;18:424. doi: 10.1186/s12864-017-3810-7 (PMC5450241; doi:10.1186/s12864-017-3810-7)
Supplement: Supplementary file 15 — Histograms of log2 fold change values for genes differentially expressed (FDR ≤ 0.1) at 15 dpf. Histograms of log2 fold change values for genes differentially expressed (FDR ≤ 0.1) at 15 dpf in all pairwise comparisons. Most genes are differentially expressed by 1.2–1.5 fold difference, with a much smaller number of genes DE by greater than 1.5 fold indicating a modest change to the magnitude at which most genes are DE. But compare to embryonic stages 48 hpf and 96 hpf there are many more genes DE by greater than 1.5 fold. Insets highlight genes differentially expressed at log2 fold change less than 2. (PDF 265 kb) [file 12864_2017_3810_MOESM15_ESM.pdf]

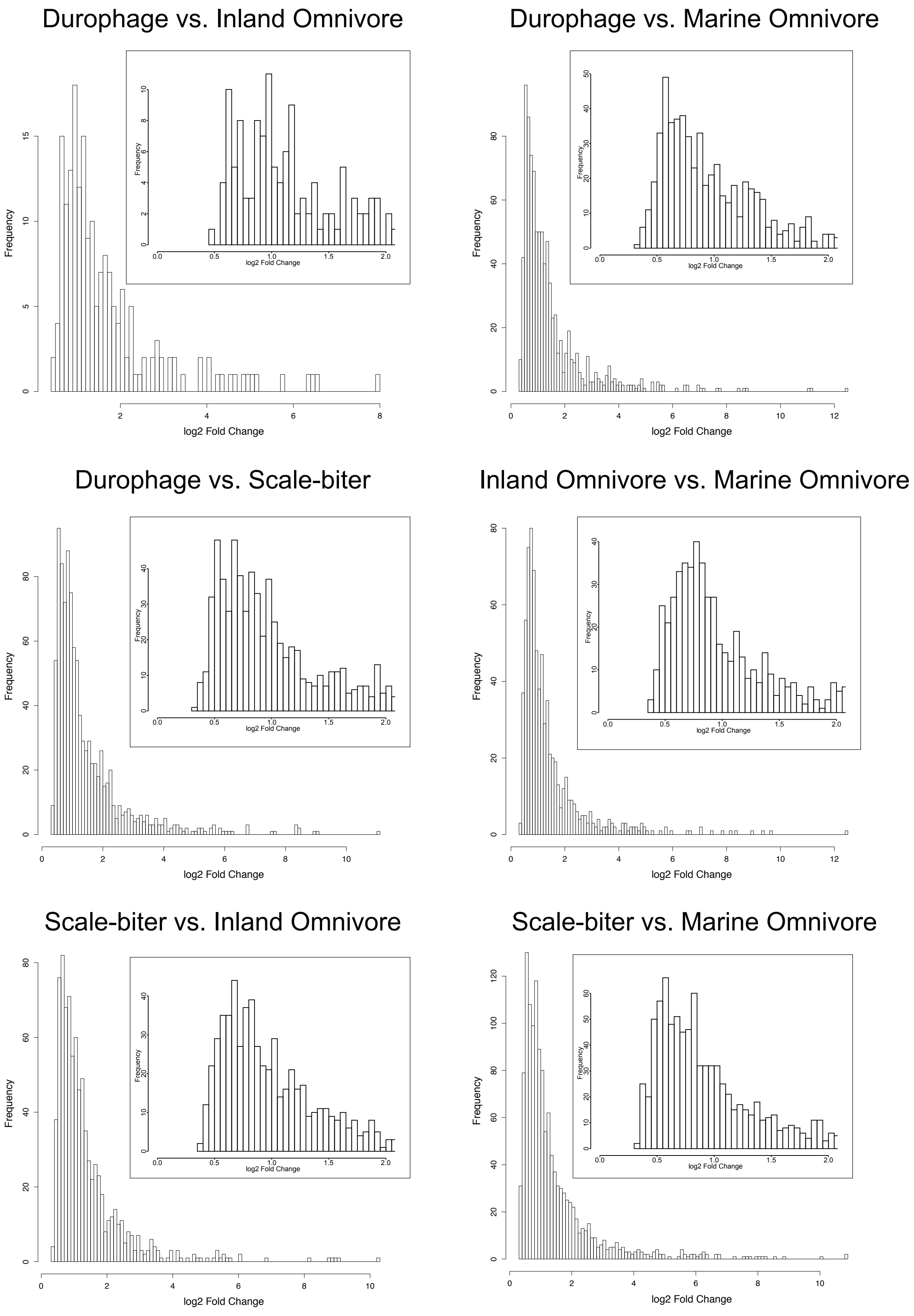
Durophage vs. Inland Omnivore
Durophage vs. Marine Omnivore
Durophage vs. Scale-biter
Inland Omnivore vs. Marine Omnivore
Scale-biter vs. Inland Omnivore
Scale-biter vs. Marine Omnivore
Frequency
log2 Fold Change

Figure S8
