# Supplementary material for: The Cyprinodon variegatus genome reveals gene expression changes underlying differences in skull morphology among closely related species
Source: BMC Genomics. 2017 May 30;18:424. doi: 10.1186/s12864-017-3810-7 (PMC5450241; doi:10.1186/s12864-017-3810-7)
Supplement: Supplementary file 16 — Heatmap of genes in both the scale-biter and durophage intersection sets. Differentially expressed genes are over- or underexpressed in a single taxon. Shown are heatmaps of all genes in both the scale-biter and durophage intersection sets at 48 hpf (A) and at 96 hpf, 8 dpf, and 15 dpf (B). Note how the data fall into four main clusters at each stage that are easily visualized by eye corresponding to genes over or underexpressed in either the scale-biter or durophage respectively. In contrast, genes are not typically differentially expressed in both the scale-biter and durophage taxa. For instance, the plot in A shows genes overexpressed in the scale-biter, and note that these same genes are similarly expressed among the other three taxa. (PDF 12001 kb) [file 12864_2017_3810_MOESM16_ESM.pdf]

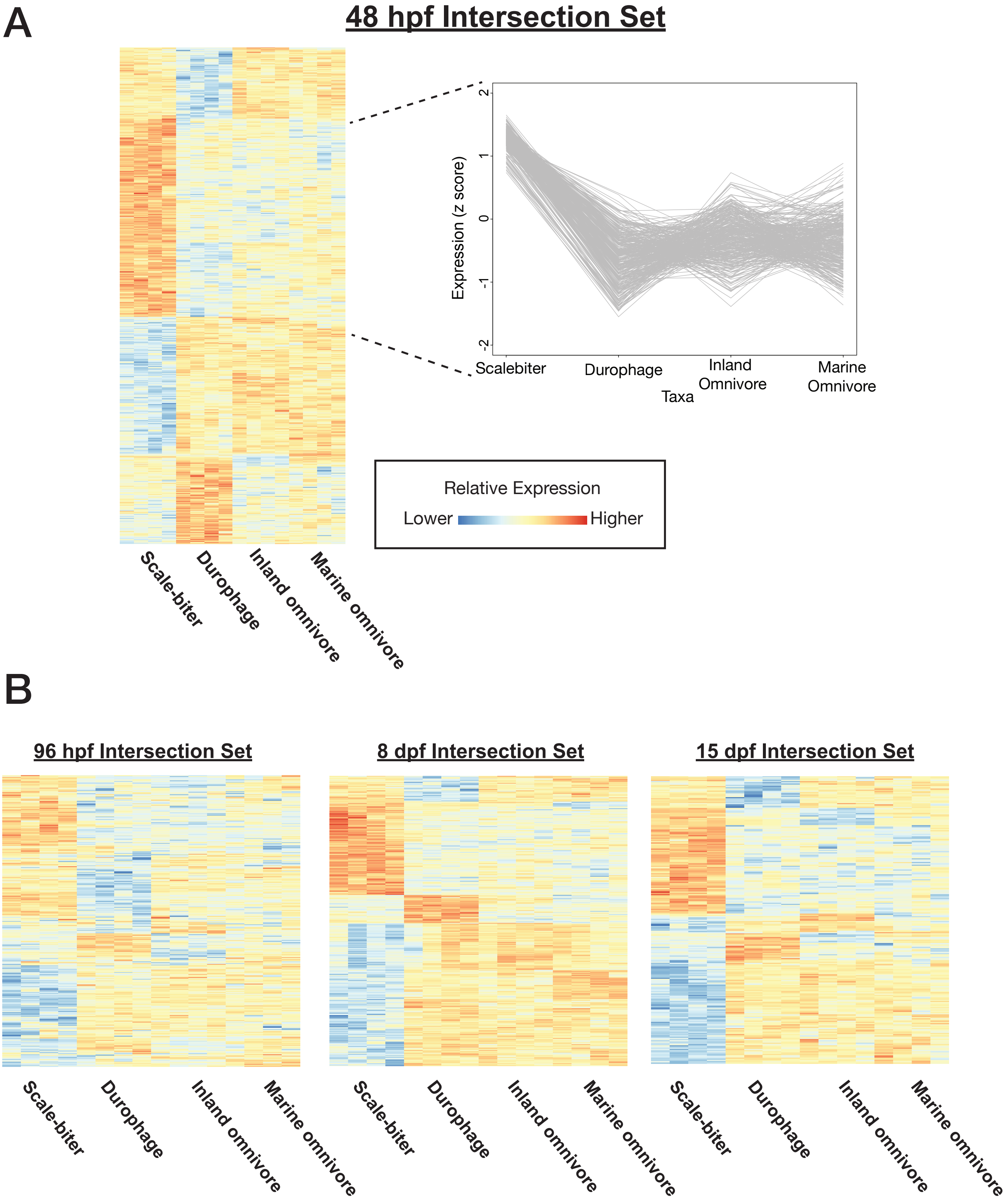
A
48 hpf Intersection Set
Expression (z score)
2
1
0
-1
-2
Scalebiter
Durophage
Inland Omnivore
Marine Omnivore
Taxa
Relative Expression
Lower
Higher
Scale-biter
Durophage
Inland omnivore
Marine omnivore
B
96 hpf Intersection Set
8 dpf Intersection Set
15 dpf Intersection Set
Scale-biter
Durophage
Inland omnivore
Marine omnivore
Scale-biter
Durophage
Inland omnivore
Marine omnivore
Scale-biter
Durophage
Inland omnivore
Marine omnivore

Figure S9
